# Infrared single-photon detection with superconducting magic-angle twisted bilayer graphene

**DOI:** 10.1126/sciadv.adp3725

**Published:** 2024-09-18

**Authors:** Giorgio Di Battista, Kin Chung Fong, Andrés Díez-Carlón, Kenji Watanabe, Takashi Taniguchi, Dmitri K. Efetov

**Affiliations:** ^1^ Fakultät für Physik, Ludwig-Maximilians-Universität, Schellingstrasse 4, München 80799, Germany.; ^2^ Quantum Engineering and Computing Group, Raytheon BBN Technologies, Cambridge, MA 02138, USA.; ^3^ Department of Physics, Harvard University, Cambridge, MA 02138, USA.; ^4^ Research Center for Functional Materials, National Institute for Materials Science, 1-1 Namiki, Tsukuba 305-0044, Japan.; ^5^ International Center for Materials Nanoarchitectonics, National Institute for Materials Science, 1-1 Namiki, Tsukuba 305-0044, Japan.; ^6^ Munich Center for Quantum Science and Technology (MCQST), München, Germany.

## Abstract

The moiré superconductor magic-angle twisted bilayer graphene (MATBG) shows exceptional properties, with an electron (hole) ensemble of only ~10^11^ carriers per square centimeter, which is five orders of magnitude lower than traditional superconductors (SCs). This results in an ultralow electronic heat capacity and a large kinetic inductance of this truly two-dimensional SC, providing record-breaking parameters for quantum sensing applications, specifically thermal sensing and single-photon detection. To fully exploit these unique superconducting properties for quantum sensing, here, we demonstrate a proof-of-principle experiment to detect single near-infrared photons by voltage biasing an MATBG device near its superconducting phase transition. We observe complete destruction of the SC state upon absorption of a single infrared photon even in a 16–square micrometer device, showcasing exceptional sensitivity. Our work offers insights into the MATBG-photon interaction and demonstrates pathways to use moiré superconductors as an exciting platform for revolutionary quantum devices and sensors.

## Teaser

The moiré superconductor magic-angle graphene offers a promising pathway for single-photon detection.

## INTRODUCTION

Superconducting materials are at the heart of advanced technologies, as they are central active elements for modern quantum computing, quantum sensing, and quantum metrology applications. In particular, nanopatterned superconducting thin films have gained attention for ultrasensitive photodetection (*1*–*3*), as these combine a low heat capacity and a sharp superconducting transition. When a photon is absorbed in such a device, it breaks Cooper pairs and generates quasiparticles above the superconducting gap, thereby introducing a change in impedance. Harnessing this mechanism, superconductor-based detectors, such as transition-edge sensors (*4*, *5*), superconducting nanowires (*6*–*8*), hot electron bolometers (*9*), kinetic inductance detectors (*10*), and Josephson junctions (*11*–*13*), are among the best photodetectors for applications demanding high sensitivity, e.g., communication, radio astronomy (*14*), quantum network (*15*), and spectroscopy (*16*).

**Fig. 1.**
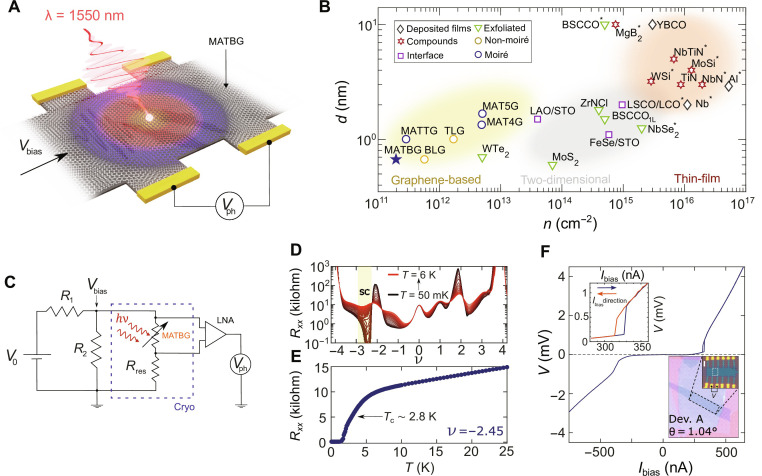
Superconducting MATBG as an ultrasensitive material for SPD. (**A**) The incident near-infrared photon breaks Cooper pairs generating a photovoltage output, *V*_ph_. (**B**) Logarithmic plot of film thickness *d* versus carrier density *n* for various superconductors (*19*, *24*–*28*, *30*, *46*–*50*). MATTG, magic-angle twisted trilayer graphene; MAT4G, magic-angle twisted four-layer graphene; MAT5G, magic-angle twisted five-layer graphene; BLG, Bernal bilayer graphene; TLG, rhombohedral trilayer graphene. The materials previously used for photodetection applications are marked with an asterisk (*3*, *6*, *18*–*20*). (**C**) Simplified circuit diagram used to measure MATBG’s photoresponse. The near-infrared photons, incident on the voltage-biased MATBG (*R*_1_ = 1 megohm, *R*_2_ = 1 kilohm, and *R*_res_ = 53 kilohm for device A), induce voltage spikes in the detector (sketched as a variable resistor) that are recorded using an oscilloscope or an analog-to-digital converter. LNA, low-noise amplifier. (**D**) Longitudinal resistance *R_xx_* of device A (θ = 1.04°) as a function of the filling factor ν for successive temperatures *T* ranging from 50 mK to 6 K. A pronounced superconducting state is observed for −3 < ν < −2. (**E**) *R_xx_* versus *T* at the optimal doping of ν = −2.45. (**F**) *I*-*V* curve measured at the optimal doping, displaying a hysteretic behavior with respect to the sweeping direction of the bias current, highlighted in the top inset. In the bottom inset, the optical image of the MATBG device. In white dashed box, the measured area is *A* ~ 16 μm^2^. Scale bar, 3 μm.

Two-dimensional superconductors offer a unique approach to single-photon detection (SPD), due to their reduced electronic heat capacity and electron-phonon coupling, leading to a large temperature rise of the electron ensemble upon absorption of single photons (*17*–*21*). It has been recently found that the flat electronic band structure produced by stacking two layers of graphene twisted at a “magic” angle leads to a unique superconducting phase (*22*, *23*). The discovery has now expanded into an entire family of graphene-based superconductors (*24*–*27*), not only sparking intense investigations to understand the fundamental physics of their alleged unconventional superconducting states but also prompting exploration of their potential applications (*28*–*30*). Specifically, moiré superconductor magic-angle twisted bilayer graphene (MATBG) can be a promising material for kinetic inductance detectors (*10*). When a single photon is absorbed in an MATBG that is embedded in a resonator, its kinetic inductance will increase, causing a shift of the resonance frequency, δ*f*. This shift is proportional to the ratio between the density of the generated quasiparticles (δ*n*_qp_) to Cooper pairs (*n*_s_): δ*f* ~ δ*n*_qp_/*n*_s_ (*10*) and would be large for MATBG because we expect a low *n*_s_ as the intrinsic moiré superlattice of MATBG has a record-low carrier density of *n* ~ 10^11^ cm^−2^, which is ~5 orders of magnitude lower than conventional superconductors (Fig. 1B). In this material, even a minute amount of quasiparticles generated by a single low-energy photon can induce a substantial change in the kinetic inductance, opening a promising avenue to extend SPD across a broader spectral range.

In this study, we take the first step to develop an SPD based on superconducting MATBG and perform a proof-of-principle experiment to demonstrate the capability of detecting single photons. We illuminate the device at millikelvin temperatures with a highly attenuated 1550-nm laser source and monitor the induced photovoltage (*V*_ph_), as shown in the schematic drawing of Fig. 1A.

## RESULTS

The optical image (inset of Fig. 1F) shows a typical device. The van der Waals stack consists of two graphene sheets rotated at a global twist angle of ~1.1° encapsulated into insulating hexagonal boron nitride (hBN) layers. The metallic graphite gate underneath the heterostructure is used to electrostatically tune the carrier concentration in the MATBG by applying an external gate voltage. Figure 1D shows the four-terminal longitudinal resistance *R_xx_* of device A (θ *=* 1.04° ± 0.02) as a function of the moiré filling factor ν (filling of electrons per moiré unit cell) for temperatures ranging from *T* = 50 mK up to *T* = 6 K. At electrostatic doping levels corresponding to the half-filling of the moiré unit cell (ν = −2), we observe an insulating state flanked by a pronounced superconducting dome (*22*). In Fig. 1E, the measurement of *R_xx_* versus *T* at the optimal doping of ν = −2.45 reveals a superconducting transition with a normal state resistance of ~10 kilohm and a critical temperature of *T*_c_ ~ 2.8 K, calculated as 50% of its normal state resistance (see the Supplementary Materials for a complete transport characterization).

Figure 1F plots the *I*-*V* characteristic of the superconducting state of device A measured in a four-terminal current-biased scheme at *T* = 35 mK. Here, we observe a clear hysteretic behavior with respect to the sweeping direction of the bias current *I*_bias_, characterized by Δ*I = I*_c_ − *I*_r_ *~* 15 nA, where *I*_c_ and *I*_r_ are the switching and retrapping current, respectively (see the Supplementary Materials). The hysteresis loops are ubiquitous in MATBG (*31*), potentially due to a current-induced self-heating hotspot (*32*, *33*) when the MATBG is in the normal state.

### Photoresponse measurements

We can bias our device near the normal-superconductor transition to enable SPD. When the photon is absorbed, it breaks Cooper pairs and produces a voltage output. To prevent the detector to “latch” permanently into a stable resistive state where it no longer detects photons, we implement a self-reset circuitry (Fig. 1C). The circuit is constituted by a voltage divider with load resistor *R*_2_ < < *R*_res_ + *R*_MATBG_. Here, *R*_res_ is a residual resistance (arising from the contact resistance and the metallic leads), and *R*_MATBG_ is the four-terminal resistance of the device active region, which is sketched as a variable resistor. The voltage bias scheme maintains a constant voltage across the source and drain contacts of the device (*V*_bias_). In this way, the increase in resistance induced by the switching of the MATBG detector in the normal state diverts part of the current into the load resistor *R*_2_, reducing the current flowing in the detector and, consequently, the Joule heating, analogously to an electrothermal feedback (*34*, *35*). Once the current is reduced, the detector returns to the superconducting state. As illustrated in Fig. 1C, the voltage probes in the four-terminal scheme are connected to a room-temperature low-noise amplifier, and the output of which is fed to an oscilloscope or an analog-to-digital converter to measure the voltage over time induced by the photons. When the MATBG transitions into the resistive state upon photon absorption, we register a spike in *V*_ph_, the detector resets itself, and we can measure the statistics of counts as a function of bias voltage, laser power, and temperature.

To perform the photoresponse measurements, we mounted the MATBG device in a dilution refrigerator and provided optical excitation with a 1550-nm laser diode. The beam was collimated in the sample space (~4-mm spot diameter) allowing illumination of the entire device area. The incident laser power was then controlled using a programmable optical attenuator (see Materials and Methods). In our experimental setup, both bias and readout leads were heavily filtered to ensure millikelvin electron temperature at the sample stage. The constrained electrical bandwidth available in the experiment imposes limitations on the maximum detector count rate and the speed of the reset circuitry but still allows to properly studying the statistics of the photoinduced counts. We extensively describe the optoelectronic setup used in our experiment and the method used to register the counts in the Supplementary Materials.

**Fig. 2.**
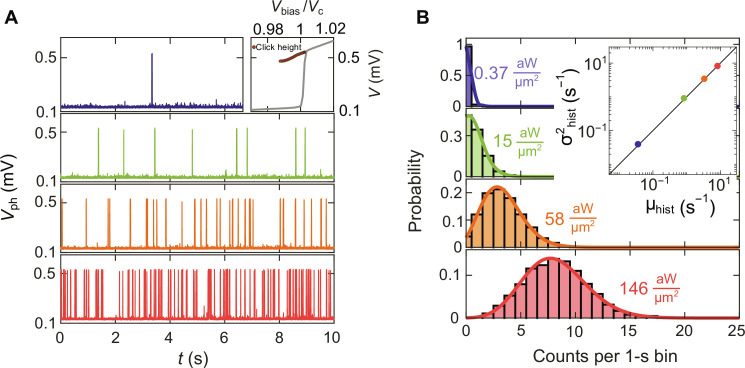
Statistics of the light-induced clicks. (**A**) Raw photovoltage time traces, *V*_ph_(*t*), measured at increasing laser powers for λ = 1550 nm. Top right inset: Average click height measured as a function of *V*_bias_. The click heights are overlaid on the *I*-*V* curve, measured in the configuration described in Fig. 1C. (**B**) Histograms of counts in 1-s bins for the same laser powers in (A) measured over ~10^3^-s time window. The inset shows that the extracted variance of counts, σ^2^_hist_, equals to its mean μ_hist_. The agreement with the Poisson statistic is confirmed by plotting the Poisson distribution on top of the histograms, with the extracted μ_hist_ and σ^2^_hist_ (solid lines).

Figure 2A illustrates examples of the photovoltage traces *V*_ph_(*t*), measured over time across the MATBG detector when exposed to the laser beam radiation in the configuration described in Fig. 1C. We observe voltage spikes, emerging as we increase the incident laser power, which we attribute to photoinduced switching events from the superconducting to the normal state. To confirm the origin of the voltage spikes, which are the “clicks” of our detector, we investigate their stochastic nature by producing histograms of counts with 1-s bins and extracting the mean (μ_hist_) and variance (σ^2^_hist_) of the sampling distribution (*36*). As reported in the inset of Fig. 2B, for all the histograms, the mean equals the variance, as prescribed by a Poisson process. We further demonstrate the agreement with this statistic by plotting the Poisson distribution on top of the histograms, with the extracted μ_hist_ and σ^2^_hist_ (solid lines). The excellent agreement between the experimentally registered counts and the statistical model confirms that our observation is compatible with the photon shot noise generated by the highly attenuated continuous wave (CW) laser source.

In addition, we examine the average click height as a function of the bias voltage, *V*_bias_. These results are overlaid on the *I*-*V* curve (top inset in Fig. 2A), which was measured in the configuration described in Fig. 1C. We find that the generated photovoltage closely matches the voltage in the normal state across all explored bias voltages: *V*_ph_(*V*_bias_) ≈ *V*(*V*_bias_). This observation indicates that the incident photons induce a complete transition of the MATBG detector from its superconducting state to the normal state.

### Single-photon sensitivity by superconducting MATBG

**Fig. 3.**
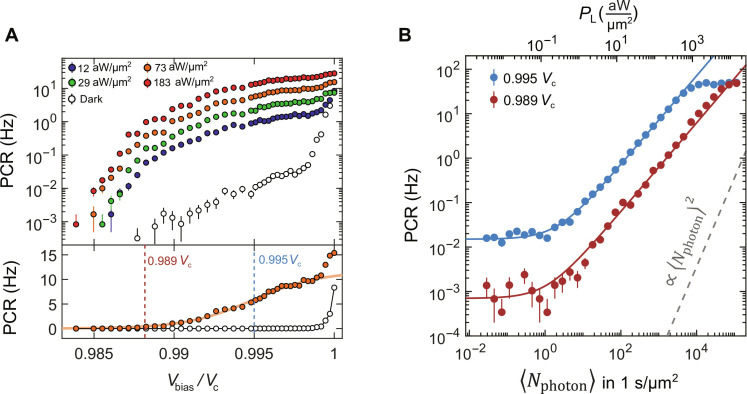
Single-photon sensitivity by superconducting MATBG. (**A**) Top: PCR as a function of voltage bias *V*_bias_ for four different laser powers (filled dots) and in the dark (empty dots). Bottom: PCR versus *V*_bias_ for *P*_L_ = 73 aW/μm^2^ on a linear scale. The orange line is a fit with a sigmoid function. The PCR shows a tendency to saturation at ~0.997 *V*_c_. The vertical dashed lines are the bias points at which we performed the PCR versus *P*_L_ measurements reported in (B). (**B**) PCR versus average incident photon number 〈*N*_photon_〉 in a 1-s time window per square micrometer for two different bias points (*V*_bias_ = 0.995 *V*_c_ and *V*_bias_ = 0.989 *V*_c_). On the top *x* axis, the corresponding incident CW power density *P*_L_ = 〈*N*_photon_〉 ∙ *h*ν*/*τ. The solid lines are linear fits (with an offset due to dark counts), showing that the detection probability evolves linearly with 〈*N*_photon_〉. The gray dashed line depicts a quadratic power dependence.

To investigate the observed photoresponse, in Fig. 3A, we compare the photon count rate (PCR) (counts recorded per second) for different bias voltages (*V*_bias_) without light (empty dots) and with an excitation wavelength of λ = 1550 nm for different laser powers (filled dots). When the detector operates at a bias voltage far from the transition (*V*_c_), the PCR is orders of magnitude higher upon illumination than in the dark, while as *V*_bias_ approaches *V*_c_, a sudden increase in false-positive (dark) counts occurs, ultimately dominating the detector’s response. We fit the PCR versus *V*_bias_ curve under illumination with a sigmoid function (solid line in Fig. 3A, bottom) and observe that the experimental data exhibit a tendency to saturation at *V*_bias_ ~ 0.997 *V*_c_. These saturations are intrinsic to the process of SPD, rather than extrinsic, e.g., speed of the measurement circuitry (see the Supplementary Materials), and resemble the photon counts in superconducting nanowire SPDs (SNSPDs) (*7*). In SNSPDs, the saturation of the PCR as a function of current bias indicates that the internal detector efficiency (*37*), without coupling, reaches unity. Conversely, in our experiment, the PCR curve does not entirely saturate, implying that the intrinsic efficiency attained is not 100%.

We can demonstrate that the registered counts are triggered by single near-infrared photons. For this purpose, we explore how the count rate evolves as a function of the CW laser power over several orders of magnitude at two different bias points. To provide a quantitative description of the light-induced count rate, we estimate the power density incident on the MATBG (*P*_L_) in the approximation of a Gaussian beam (*38*). From *P*_L_, we can calibrate the number of incident photons per square micrometer in a time window τ as 〈*N*_photon_〉 = τ · *P*_L_*/h*ν, where *h*ν = 1.28 × 10^−19^ J is the energy of a single photon at λ = 1550 nm. Choosing τ = 5 ms, which is close to the typical detector recovery time, a laser power density of *P*_L_ = 10 aW/μm^2^ corresponds to 〈*N*_photon_〉 = 0.4 photons incident/μm^2^ in a time window of 5 ms (see the Supplementary Materials). Under illumination with a weak coherent light source (*36*), the probability of detecting *m* photons in a detection time window reduces to ~〈*N*_photon_〉*^m^* /*m*!. Figure 3B shows the PCR as a function of 〈*N*_photon_〉. The measured detection probability increases linearly with 〈*N*_photon_〉 over >3 orders of magnitude with an offset due to the dark counts, demonstrating single-photon sensitivity of the MATBG superconducting detector (*1*). As reported for other SPDs (*39*), we observe that the count rate deviates from linearity at low photon fluxes, when it enters the noise level defined by the dark counts and at high photon fluxes when it saturates because of the limited bandwidth of measurement circuitry.

The trace measured at 0.995 *V*_c_ shows the same overall behavior as the one measured at 0.989 *V*_c_, with a higher detection probability and dark count rate due to the increase in the intrinsic quantum efficiency as we approach *V*_c_. In the Supplementary Materials, we show several raw photovoltage time traces at different *V*_bias_ and *P*_L_ values from which we extract the PCR reported in Fig. 3 and detail the method used to register the counts in the MATBG detector. For completeness, we also demonstrate single-photon sensitivity under pulsed light excitation (see the Supplementary Materials).

### Detector performance at higher temperatures

**Fig. 4.**
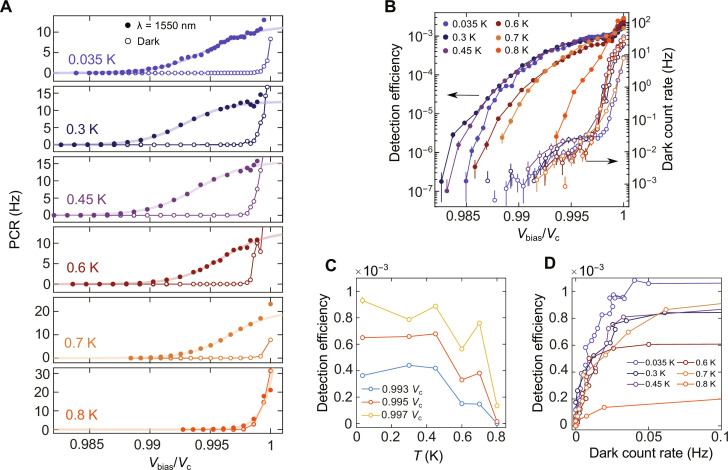
Detector performance at higher temperatures. (**A**) PCR as a function of voltage bias *V*_bias_ and temperature *T* upon illumination (filled dots) and in the dark (empty dots). The continuous lines are fit with the sigmoid function. (**B**) Filled markers indicate detection efficiency versus *V*_bias_ at different temperatures. Empty markers indicate dark count rate versus *V*_bias_ at different temperatures. The detection efficiency is defined as the ratio of counts detected per second to photons incident per second in the area (*A* ~ 16 μm^2^) between the two voltage probes. (**C**) Detection efficiency versus *T* for three different bias points extracted from the sigmoidal fit of (A). (**D**) Trade-off between detection efficiency and dark count rate for different temperatures.

To provide further insights on the photodetection mechanism in MATBG, in Fig. 4A, we present the PCR versus *V*_bias_ at six different temperatures ranging from 35 to 800 mK, both with and without laser excitation (filled and empty dots, respectively). The PCRs with illumination are consistent with the sigmoid function and exhibit a tendency to saturation for *V*_bias_ *~* 0.997 *V*_c_. Using the linear scaling of the PCR with laser power (fig. S15), we confirm the SPD from our MATBG device up to ~0.7 K. The single-photon PCR eventually vanishes when temperature rises up to 0.8 K, at which the dark count dominates the PCR. Figure 4B plots the SPD efficiency as a function of *V*_bias_ and the dark count rate (PCR without illumination) at various temperatures. Here, the efficiency is defined as the ratio of counts detected per second to photons incident per second in the area (*A* ~ 16 μm^2^) between the two voltage probes (white dashed box in the optical image of Fig. 1F). The dark count rate (right-hand side of the *y* axis) exhibits two distinct *V*_bias_ dependence above and below *V*_bias_ = 0.998 *V*_c_. When *V*_bias_ > 0.998 *V*_c_, a sharp increase in dark counts occurs. This justifies the abrupt rise of PCR under illumination when *V*_bias_ ~ *V*_c_. When *V*_bias_ < 0.998 *V*_c_, the dark counts rise gradually because of background photons coupling through the optical fiber connected at the room-temperature optical port (*40*). The detection efficiency on the left-hand side of the *y* axis is at maximum and gradually decreases as the temperature rises, akin to observations in other SPDs (*37*, *40*). To further investigate this trend, we extract the efficiency at three different *V*_bias_ values from the sigmoid fit of Fig. 4A and plot them against temperature in Fig. 4C. The efficiency decreases as temperature rises. We attribute this to the increase in the thermal conductance. Elevated temperatures enhance heat transfer out of the electrons (*41*), reducing the probability of latching into the resistive state by a self-heating effect. This argument is supported by thermal transport measurements in the superconducting state on the same system, which report a rapid increase in thermal conductivity within the range 35 mK < *T* < 800 mK (*30*). Figure 4D plots the trade-off between SPD efficiency and dark count rate at various temperatures to determine the optimal operating condition of our MATBG detector. We observe that the most favorable SPD performance is achieved in the plateau region, where the efficiency approaches its maximum value while maintaining a low dark count rate (*11*).

## DISCUSSION

In addition to the demonstration of SPD, our experiment offers insight to the MATBG superconductivity via its interaction with photons. Since the incident photon energy (~0.8 eV) greatly exceeds the flat bands’ width (~10 meV) (*42*) and the superconducting gap’s size (~1 meV) (*30*, *43*), we can approximate MATBG’s absorption to be the same as bilayer graphene. Using the transfer matrix formalism (see the Supplementary Materials), we estimate ~5.3%. With the measured PCR at the saturation plateau in Fig. 4B, we estimate the internal efficiency of our SPD as ~10^−3^/0.053 to 0.019. Two factors can limit the internal efficiency in our setup. First, the effective area of the MATBG contributing to the photoresponse could be much smaller than the entire area of the device. This argument would agree with the measurements of twist angle inhomogeneity by local probes techniques on MATBG (*44*, *45*). Twisted moiré materials are characterized by an intrinsic disorder due to the local variation of twist angle within the same sample. Because of the relaxation of the lattice structure, the atoms between the two twisted layers of graphene may not align to the same global twist angle, resulting in a narrower superconducting area in MATBG. If we assume an internal efficiency of ~1 at the saturation plateau, then we can estimate a lower limit for the effective superconducting area contributing to the photoresponse as *A*_eff_ ~ 0.019 and *A* ≈ 0.3 μm^2^. If the superconducting channel fully percolates between the two voltage probes, which are spaced about 3 μm apart, then we estimate the channel’s width to be approximately 100 nm. This scenario would be similar to the SNSPDs in which the absorbed photon generates a resistive domain of quasiparticles to produce a readout signal (*6*). Unlike SNSPDs, however, the hotspot in MATBG would expand with self-sustained Joule heating, leading to a complete breakdown of superconductivity across the whole superconducting path, as observed in the inset of Fig. 2A. Hence, heat dissipation, which reduces the latching probability, could be the second factor limiting the internal efficiency, as supported by the temperature-dependent data in Fig. 4C. Future applications may explore SPD readout mechanisms that do not involve a complete switching of the entire MATBG device into the normal state and could use more sensitive probes to monitor the light-induced changes in voltage or kinetic inductance (*10*).

In conclusion, our experimental work has successfully demonstrated that the superconducting state of MATBG can be used to detect single near-infrared photons. This result strongly motivates further investigation to extend single-photon capability to lower energies using MATBG and other low-carrier density graphene-based superconductors (*24*–*27*). Pursuing this route necessitates further research effort to understand the intricate interplay between incident photons and these alleged unconventional superconducting phases. Our investigation has contributed important insights into the physical process underlying the observed MATBG’s photoresponse. These insights will play a pivotal role in the development of theoretical models and in the design of innovative quantum devices that exploit the unique characteristics of these materials, ultimately advancing the field of quantum technology.

## MATERIALS AND METHODS

### Device fabrication

The MATBG devices were fabricated using the “cut-and-stack” technique. A stamp made of propylene carbonate and polydimethylsiloxane was prepared and mounted on a glass slide. The stamp was used to pick up the top layer of hBN. The hBN layer was then used to pick up the two halves of graphene, which had been precut using an atomic force microscopy tip. The graphene halves were carefully rotated to a target twist angle of 1.1°. To complete the heterostructure, the entire stack was fully encapsulated with a bottom hBN layer. A graphite layer was added at the bottom of the stack, serving as a local backgate for the device. The full stack was then deposited onto a Si/SiO_2_ chip and etched into a Hall bar geometry. Last, edge contacts made of Cr/Au (5/50 nm) were evaporated onto the device to establish electrical connections.

### Photoresponse measurements

To perform the photoresponse measurements, we placed the device on the cold finger of a dilution refrigerator (BlueFors-SD250), housed in a gold-coated oxygen-free copper box. The dilution refrigerator (base temperature of 35 mK) was optimized for low-frequency transport of low-carrier density two-dimensional superconducting materials. A two-stage RC low-pass filter was mounted at the 1-K still plate of the dilution refrigerator combined with an additional radio frequency filter mounted at the mixing chamber stage to ensure millikelvin electron temperature and reject high-frequency noise (fig. S6). The overall bandwidth of the readout was <1 kHz (fig. S9). In the SPD experiment described in Fig. 1C, the bias voltage was applied at the source contact with a voltage generator (Keithley 2400) in series with a 1/1000 voltage divider (*R*_1_ = 1 megohm and *R*_2_ = 1 kilohm for device A). The voltage probes were connected to a room-temperature 1-MHz-bandwidth low-noise amplifier (SR-560). A room-temperature low-pass filter with a sharp cutoff at ~1 to 10 kHz was used for rejecting white noise outside of the readout bandwidth. The amplified signal was fed to a sampling oscilloscope with variable bandwidth up to 600 MHz (UHF-Scope Zurich Instrument) or a 100-kHz-bandwidth analog-to-digital converter (UHF-Aux In Zurich Instrument). The optical excitation was provided by a 1550-nm laser diode (Taiko PDL M1). The light was fed into the dilution refrigerator to the MATBG detector, through a single-mode optical fiber coupled with a collimator mounted few centimeters on top of the sample space, allowing illumination of the entire device area of ~4-mm spot diameter (fig. S7). To control the incident laser power, a programmable optical attenuator (JGR OA5 l) was used, enabling precise adjustment over several orders of magnitude. Extensive description and schematics of the optoelectronic setup used in the experiment are available in the Supplementary Materials.

### Transport measurements

The longitudinal resistance *R_xx_* was measured using standard low-frequency lock-in techniques (Stanford Research SR860). To control the carrier density, a voltage was applied to the graphite metallic gate using a Keithley 2400 voltage source connected in series with a 100-megohm resistor. For measuring the current-biased *I*-*V* curves in the four-terminal configuration, the bias current was supplied at the source contact using a voltage generator (Keithley 2400) connected in series with a bias resistor of 10 megohm. The voltage probes were connected to a room-temperature low-noise preamplifier (SR-560), and the output signal was measured using a digital multimeter (Keithley 2700).

## Supplementary Materials

### This PDF file includes:

Supplementary Text Figs. S1 to S20 References
