# Supplementary material for: Infrared single-photon detection with superconducting magic-angle twisted bilayer graphene
Source: Sci Adv. 2024 Sep 18;10(38):eadp3725. doi: 10.1126/sciadv.adp3725 (PMC11409955; doi:10.1126/sciadv.adp3725)
Supplement: Supplementary file 1 — Supplementary Text Figs. S1 to S20 References [file sciadv.adp3725_sm.pdf]

Science Advances
AAAS

Supplementary Materials for

Infrared single-photon detection with superconducting magic-angle twisted bilayer graphene

Giorgio Di Battista *et al.*

Corresponding author: Dmitri K. Efetov, dmitri.efetov@lmu.de

Sci. Adv. **10**, eadp3725 (2024)
DOI: 10.1126/sciadv.adp3725

This PDF file includes:

Supplementary Text
Figs. S1 to S20
References

Extended transport characterization for device A

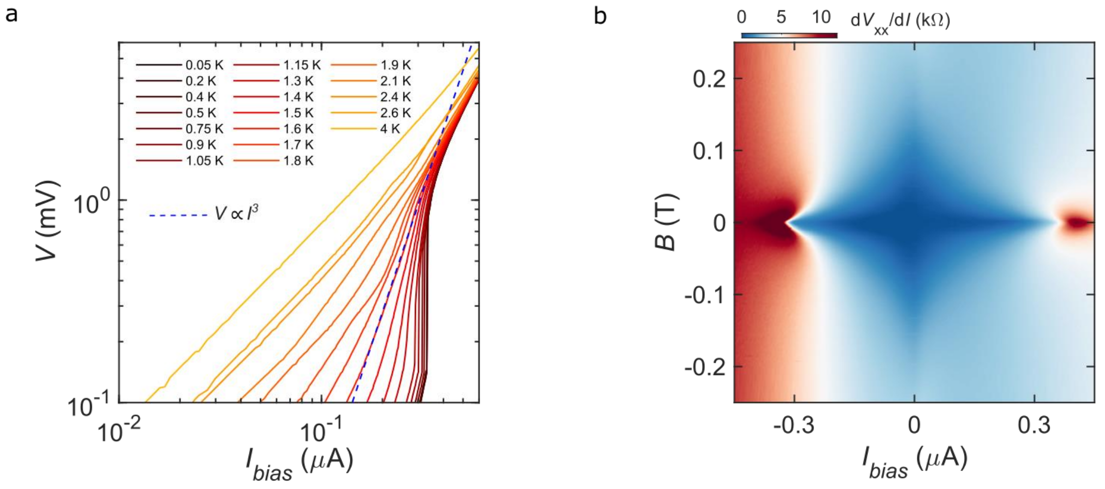

Fig. S1.| **Full characterization of the superconducting state of device A at *ν* = -2.45. (a)**. Current-voltage (*I*-*V*) curves measured at different temperatures and plotted in logarithmic scale. The logarithmic scale helps in determining the Berezinskii-Kosterlitz-Thouless transition temperature ($T_{BKT} \sim 1.6$ K) by fitting the data to a power law $V \propto I^3$ (represented by the blue dashed line). **(b)** Differential resistance dV_{xx}/dI as a function of bias current I_{bias} and out-of-plane magnetic field B for ν=-2.45. The ac excitation current used for this measurement is I_{ac} = 2 nA. The superconducting state is completely smeared out by magnetic field at $B \sim 150$ mT.

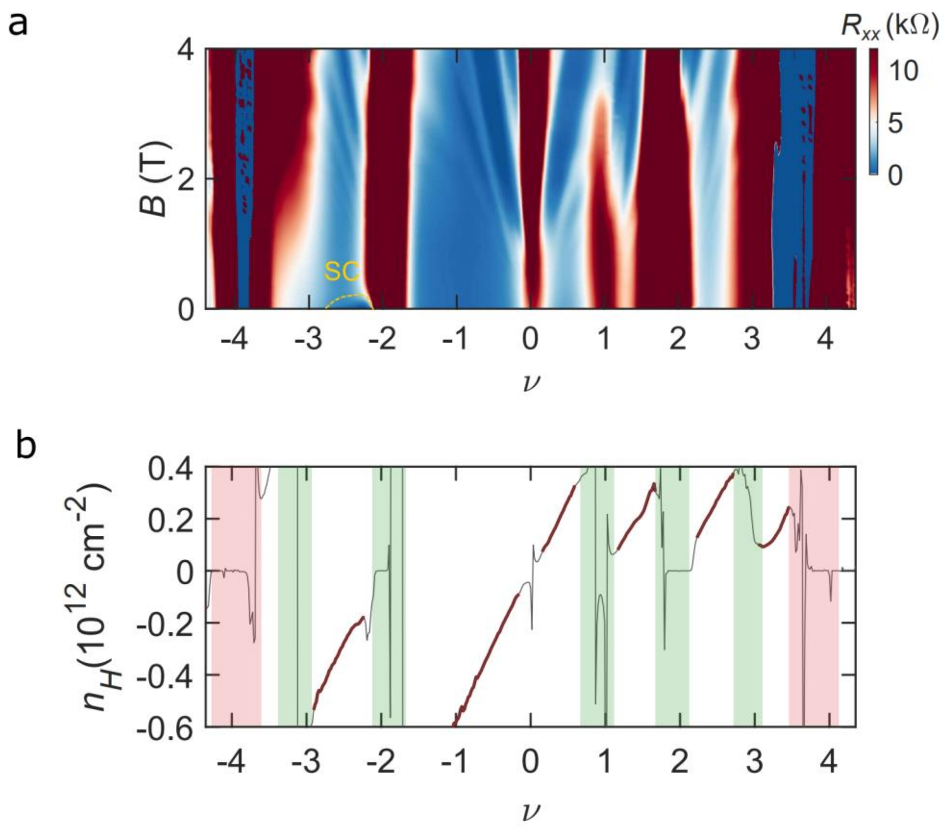

Fig. S2.| **Transport characterization with out-of-plane magnetic field for device A. (a)** Landau fan diagram measured at 35 mK with an excitation current of 10 nA and an applied out-of-plane magnetic field *B* ranging from 0 to 4 T. **(b)** Low-field Hall density measurements. The light-gray line trace shows the Hall carrier density n_H versus the moiré band filling factor ν measured at 500 mT. The light green stripes indicate the position of correlated states while the light red ones the band insulating states. The thick red lines delineate the regions where the Hall carrier density exhibits a linear relationship with the filling factor. Notably, at the integer fillings corresponding to the correlated states, we observe resets of the Hall carrier density, resulting in the presence of extremely low carriers involved in the conduction process. Specifically, for the doping used in the single-photon detection experiments (ν between -2 and -3) we expect a carrier density $n_H \sim 10^{11}$cm^{-2}.

Hysteretic *I-V* curves in superconducting MATBG devices

For this project we have produced several MATBG superconducting devices with the 'cut-and-stack' procedure described in the methods section. Among all the superconducting MATBG we have selected 3 devices which featured sharp superconducting transitions with hysteretic *I-V* characteristics and investigated their photoresponse.

Device A and C have a single bottom graphite back gate, while device B has an additional graphite top-gate which was picked up at the first step of the stacking process. The global twist angles measured from transport data for device A, B and C are $\theta = 1.04°$, $\theta = 1.03°$ and $\theta = 1.16°$ respectively.

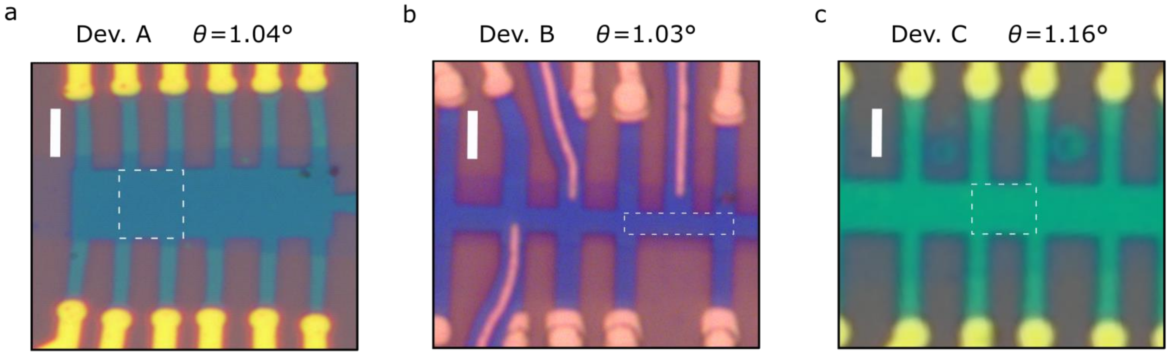

Fig. S3.| **Optical images of the measured devices.** The scale bar in all the images is 3 µm. Device A and C have a single bottom graphite back-gate while device B has a double graphite gate.

As detailed in the main text, we attribute the presence of a hysteretic loop in the *I-V* curves to a current-induced self-heating hotspot when the MATBG is in the normal state. In Fig. S4 we show the *I-V* curves of devices A, B and C at different temperatures and gate voltages (corresponding to different carrier densities). In all devices the hysteresis loop disappears for temperatures T ~ 1 K. We also point out that the applied gate voltage provides high tunability on the critical current and on the width of the hysteretic loop. This represents an important tuning knob to engineer and design the electronic circuit.

In order to control the hysteretic loop in the MATBG device, we implement the voltage-biased scheme detailed in Fig. 1c of the main text. In the voltage-biased scheme, the increase of resistance induced when the MATBG detector is in the normal state, reduces the current flowing in it and brings the device back to the superconducting state. In Fig. S5 we observe the *I*-*V* curves for device A, B and C measured in the current-biased scheme (top) and in the voltage-biased scheme (bottom). We notice that in all devices the hysteresis loop present in the current-biased scheme is completely closed in the voltage-biased one. The simple reset circuitry described here, prevents permanent 'latching' of the detector in the normal state and allows us to reset it after photo-absorption.

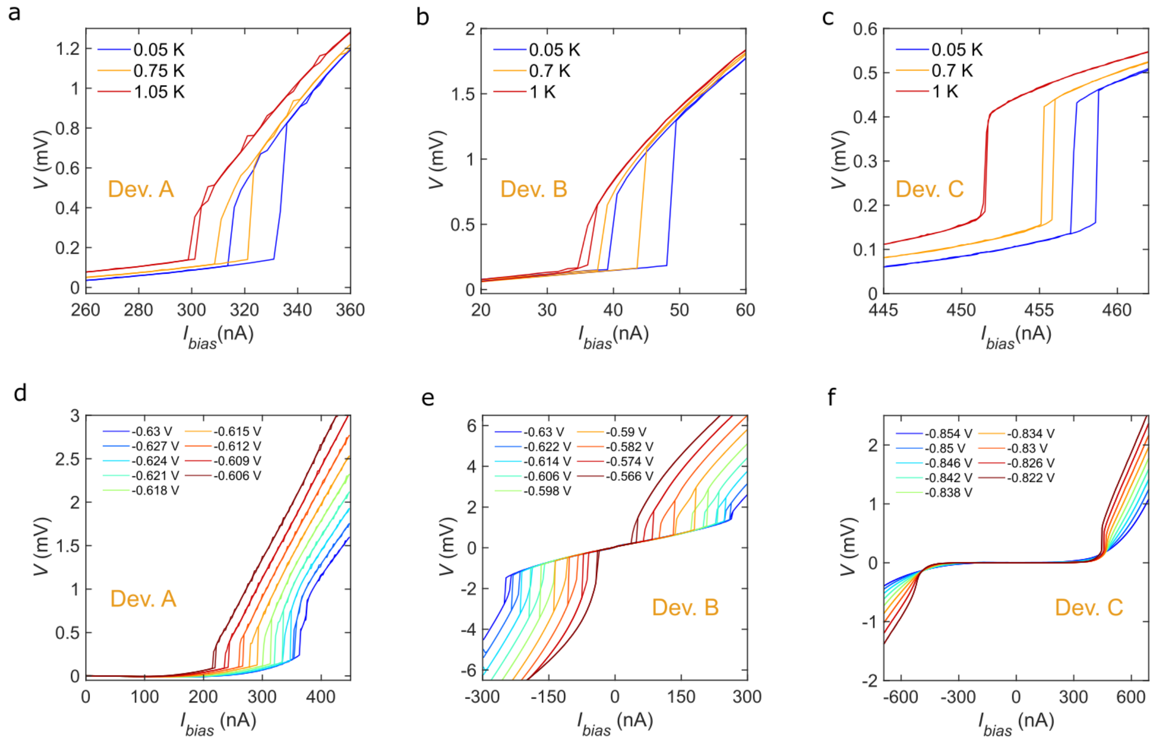

Fig. S4.| **Current-biased *I*-*V* curves at different temperatures and gate voltages. (a)-(c)** Current-biased *I*-*V* curves at 3 different temperatures for device A, B and C respectively. The *I*-*V* curves are measured at the doping (gate voltages) used for photodetection which are -0.620 V, -0.566V and -0.8257 V for device A, B and C respectively. In all the 3 devices the hysteresis loop disappears for temperatures ~ 1 K. **(d)-(f)** Current-biased *I*-*V* curves at different gate voltages (carrier densities) and T = 35 mK within the superconducting dome for device A, B and C respectively.

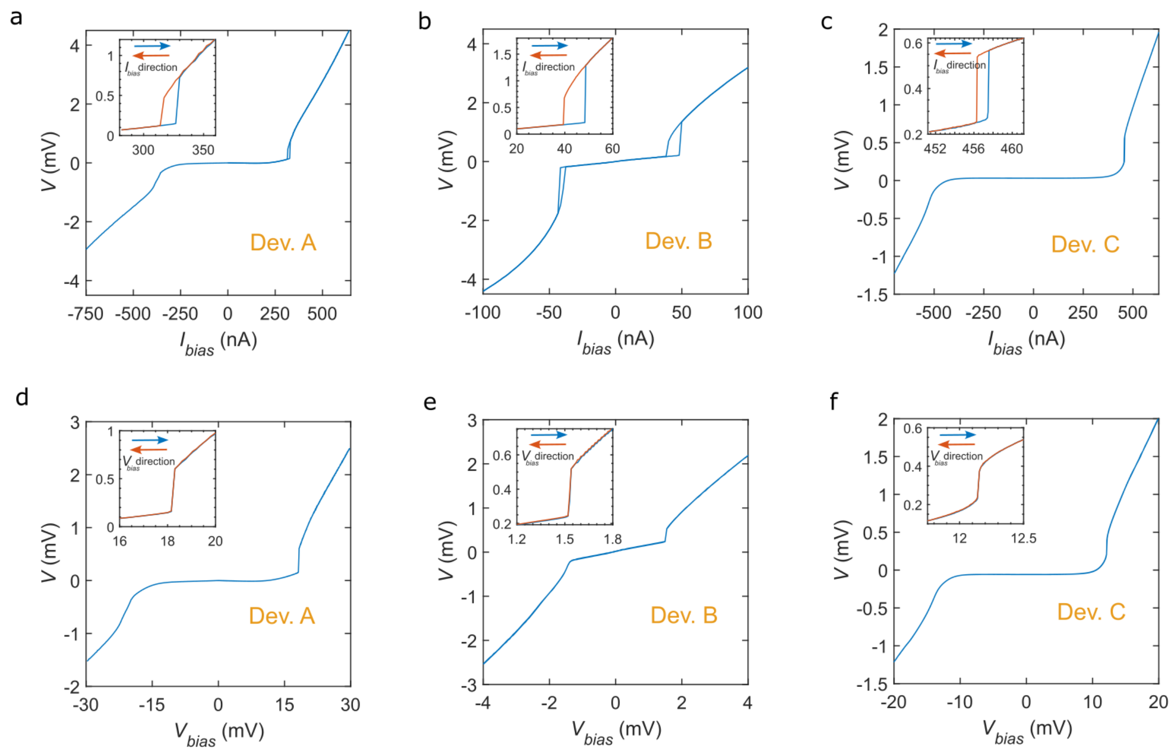

Fig. S5.| **Current-biased and voltage-biased *I*-*V* curves. (a)-(c)** Current-biased *I*-*V* curves for device A, B and C. The bias current is provided by a voltage source in series with a 10 Megaohm resistor. The *I*-*V* curves are measured at the doping used for photodetection which are -0.620 V, -0.566V and -0.8257 V for device A, B and C respectively. **(d)-(f)** Voltage-biased *I*-*V* curves for device A, B and C measured at the same doping. The bias voltage is provided by a voltage source in series with a 1/1000 voltage divider. As described in the main text the load resistor is much smaller than the residual resistance arising from the contact resistance and the metallic leads ($R_2 << R_{res.}$). R_1 = 1 Megaohm, R_2 = 1 kilohm for device A and B while R_1 = 100 kilohm, R_2 = 100 ohm for device C.

Optoelectronic setup

As explained in the Methods section an illustrated in Fig. S6, to perform the photoresponse measurements we placed the device in a dilution refrigerator and provided optical excitation with a 1550-nm laser diode coupled through a telecom single-mode optical fiber.

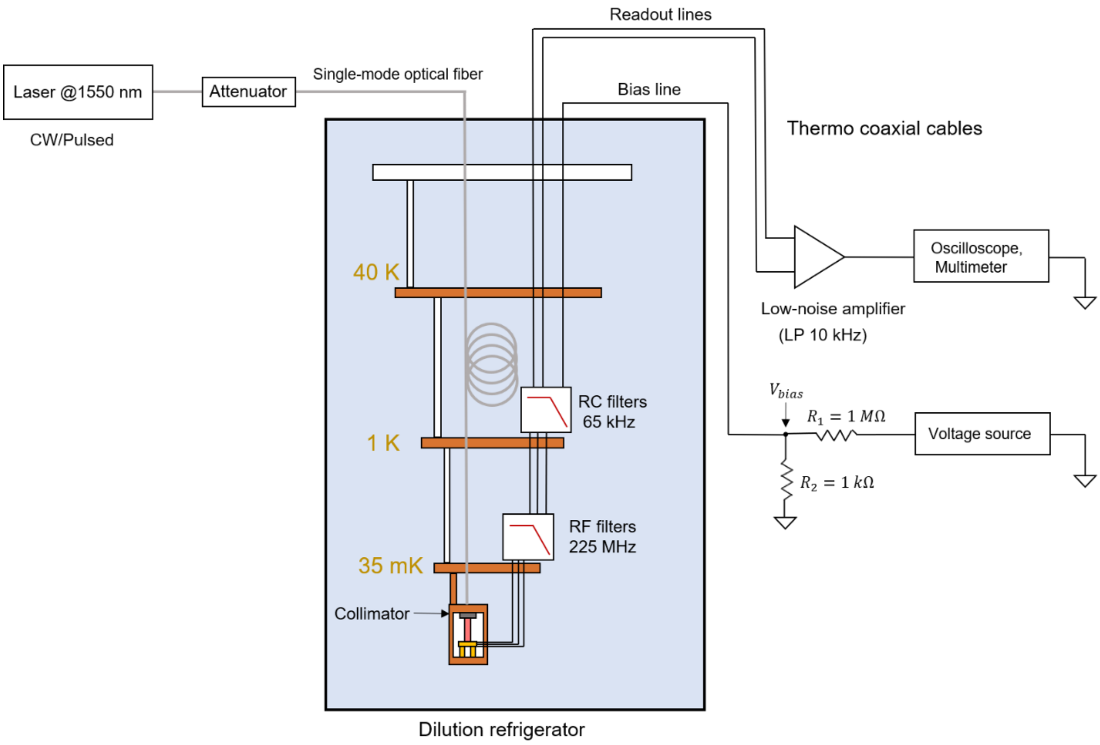

Fig. S6.| **Optoelectronic setup.** Schematics of the optoelectronic setup employed to measure the photoresponse in the MATBG superconducting detector.

Beam profile at the sample stage

As shown in Fig. S7, in our setup we couple a telecom laser which emits an output power P_{out} with a single-mode optical fiber designed for 1550-nm transmission. The fiber is then connected to a laser beam coupler which provides a collimated output with beam radius $w_0 \backsim 2$ mm and Rayleigh range $z_R = \frac{\pi w_0^2}{\lambda} \sim 8$ m. In order to quantitatively describe the light-induced count rate on the MATBG detector we consider a Gaussian beam profile. In this approximation, the intensity profile I as a function of the distance from the beam center r and distance away from the end of the coupler z reads*(38)*:

$$I(r,z) = I_0 \left(\frac{w_0}{w(z)}\right)^2 e^{-2(r/w(z))^2} \qquad \text{(Eq.1)}$$

Where *w(z)* is the value of the radius at a distance z from the fiber given*(38)* by $w(z) = w_0\sqrt{1+(z/z_R)^2}$ and $I_0 = 2P_{out}/(\pi w_0^2)$ is the total irradiance coming out of the laser source imposing the Gaussian normalization condition. In Fig. S7c we simulate *w(z)* up to 1 m of distance from the fiber coupler. Since in our experimental configuration the device is around $z_0 \sim 3$ cm away from the fiber coupler ($\frac{z_0}{z_R} = 0.0037 << 1$) we can consider the beam to be collimated and replace in (Eq.1) $w(z) \simeq w_0 = 2$ mm. Since we align the device to be roughly at the center of the beam (Eq.1) reads $I(r=0, z_0) = \frac{2P_{out}}{\pi w_0^2}$.

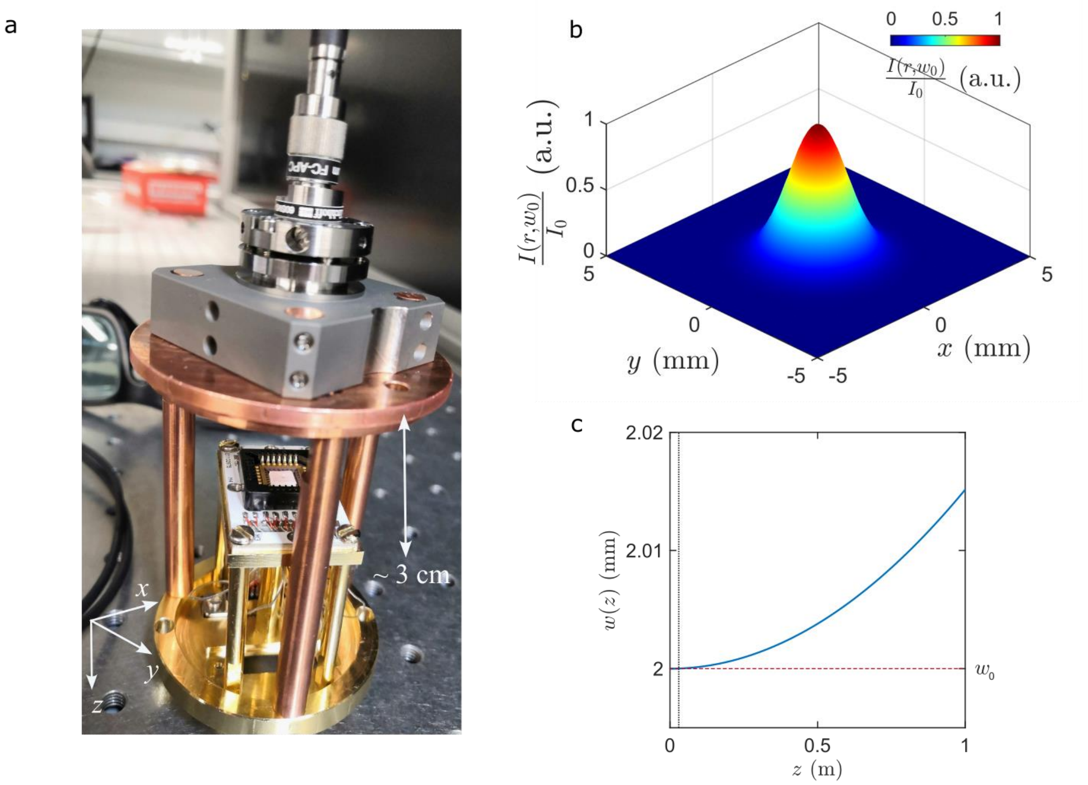

Fig. S7.| **Beam profile at the sample stage. (a)** Optical image of the experimental scheme. The single-mode optical fiber designed 1550-nm transmission is connected to a laser beam coupler which provides a collimated output. The sample is located at around 3 cm far from the fiber coupler. **(b)** 3D plot of the normalized beam intensity in the Gaussian beam approximation. **(c)** Simulation of the Gaussian beam radius *w(z)* at a distance *z* from the fiber coupler $w(z) = w_0\sqrt{1+(z/z_R)^2}$. The black vertical line is the position of the sample $z_s = 3$ cm.

Calculation of the power density incident on the MATBG device

With the considerations made in the previous section, we can calculate the power density P_L incident on the MATBG:

$$P_L = \frac{10^{-\frac{\eta}{10}} \cdot T_{fiber} \cdot P_{out}}{\frac{\pi}{2} w_0^2} \qquad \text{(Eq.2)}$$

Where P_{out} is the total power output coming out of the laser, $T_{fiber} = 0.021$ the effective transmission of the fiber and all the optical connections and η the variable attenuation (in dB) we use to control the power incident on the device. In the single-photon measurements with the CW laser source we keep the laser power constant (P_{out} = 11 µW for device A) and scan η between several order of magnitudes from 70 dB to 4 dB. For device A, a typical attenuation of 40 dB results in $P_L = 3.7\ \frac{\mathrm{aW}}{\mu\mathrm{m}^2}$.

Given P_L, the average incident photon rate per unit time τ per µm^2 $\langle N_{photon} \rangle$ reads:

$$\langle N_{photon} \rangle = \tau \cdot \frac{P_L}{h\nu} \qquad \text{(Eq.3)}$$

Where $h\nu = 1.28 \cdot 10^{-19}\ J$ is the energy of a single photon at λ = 1550 nm. For an attenuation of 40 dB and τ = 5 ms, we expect $\langle N_{photon} \rangle = 0.14$.

In pulsed experiments, by changing the laser repetition rate (f_{RR}) we can control the number of photons carried on average by each pulse μ as:

$$\mu = \frac{1}{f_{RR}} \cdot \frac{P_L}{h\nu} \cdot \int_{-l_1/2}^{+l_1/2} dx \int_{-l_2/2}^{+l_2/2} dy\ e^{-2\frac{x^2+y^2}{w_0^2}} \qquad \text{(Eq.4)}$$

Where $l_1 \sim 3$ µm and $l_2 \sim 5.3$ µm are the length and width of the area between the two voltage probes (white dashed box in the optical image of Fig. 1f). Since we assume the sample to be located at the center of the beam and $l_1, l_2 << w_0$ we can simplify (Eq.4) to:

$$\mu = \frac{1}{f_{RR}} \cdot \frac{P_L}{h\nu} \cdot l_1 l_2 \qquad \text{(Eq.5)}$$

For a typical P_{out} = 3 nW, f_{RR} = 100 Hz, and attenuation of 13 dB, we obtain $\mu = 0.62$.

It is worth noticing that the calculated values are only an upper-bound estimation because the optical alignment is not controlled accurately in the cryogenic experiment. In particular it is possible that the sample is not perfectly located in the center of the beam and that the effective incident power is lower.

Estimation of the optical absorption in the heterostructure

To investigate the effect on the substrate in the internal efficiency we employ the transfer matrix method*(53)* assuming the radiation to be linearly polarized at 1550 nm and at normal incidence. Considering the refractive indexes and the thicknesses of the layers which constitute the heterostructure (hBN = 7 nm, MATBG= 0.69 nm, hBN = 5 nm, Graphite = 2 nm, SiO2 = 285 nm) we calculate an absorption of 5.3% for the MATBG layer, which is only slightly enhanced compared to the one expected for suspended MATBG at these excitation energies (4.6%). In our experiment the substrate was not properly engineered to enhance the MATBG absorption, but we envision that this could be achieved by implementing cavities*(54)* or photonic crystals*(55)*, analogously to what was previously done in graphene.

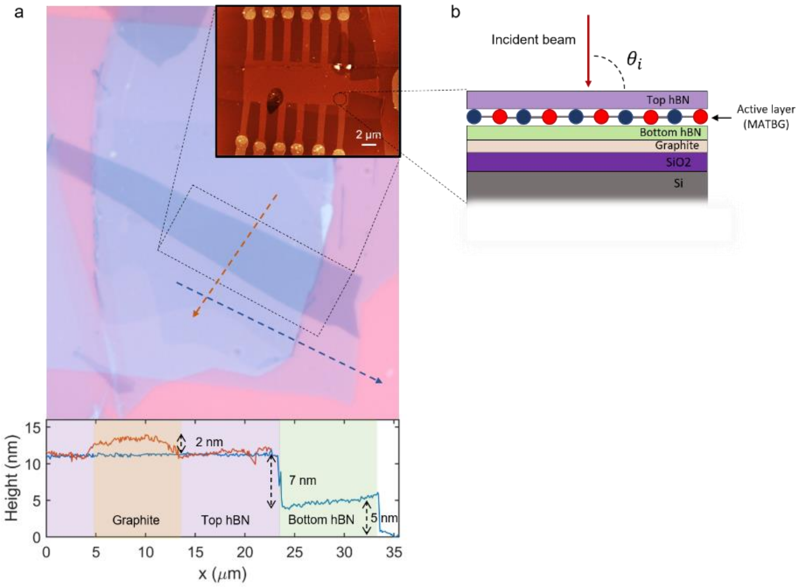

Fig. S8.| **AFM and optical micrographs for device A. (a)** The main panel is the optical image of the final hBN/MATBG/hBN/Graphite stack. The inset shows an AFM scan of the final device etched into a Hall bar geometry. The dashed square indicates the area imaged with the AFM. The lower panel shows the height profiles taken along the blue and red dashed lines from which we extract the hBNs and graphite thicknesses. **(b)** Schematic cross-section of the stack used in the transfer matrix calculations. The second layer of the heterostructure is the active one.

Effective bandwidth of the electrical readout

In this section we measure the overall bandwidth of the electronic readout available in our experiment which determines the distortion of the electrical pulses and limits the reset circuitry. Assuming that our electronic readout effectively behaves as an ideal RC low-pass circuit, we characterize its performance by determining the minimum rise time of electrical pulses and evaluating the available 3-dB bandwidth. To obtain these parameters under conditions closely resembling our single-photon detection experiment, we place a resistor (10 kilohm) into the sample space and monitor the voltage across the resistor using a 4-terminal configuration (Fig. S9b).

To measure the minimum rise time, defined as the length of time required for a signal to transition from the 10% to the 90% of the rising edge of the curve, we employ an arbitrary wave-function generator (AWG) to generate square wave pulses with a frequency of 13.3 Hz. We record the voltage across the 10 kilohm resistor with an oscilloscope (Fig. S9a, b). By analyzing the square wave pulse (Fig. S9c) we extract the minimum rise time t_r = 422 µs. From this

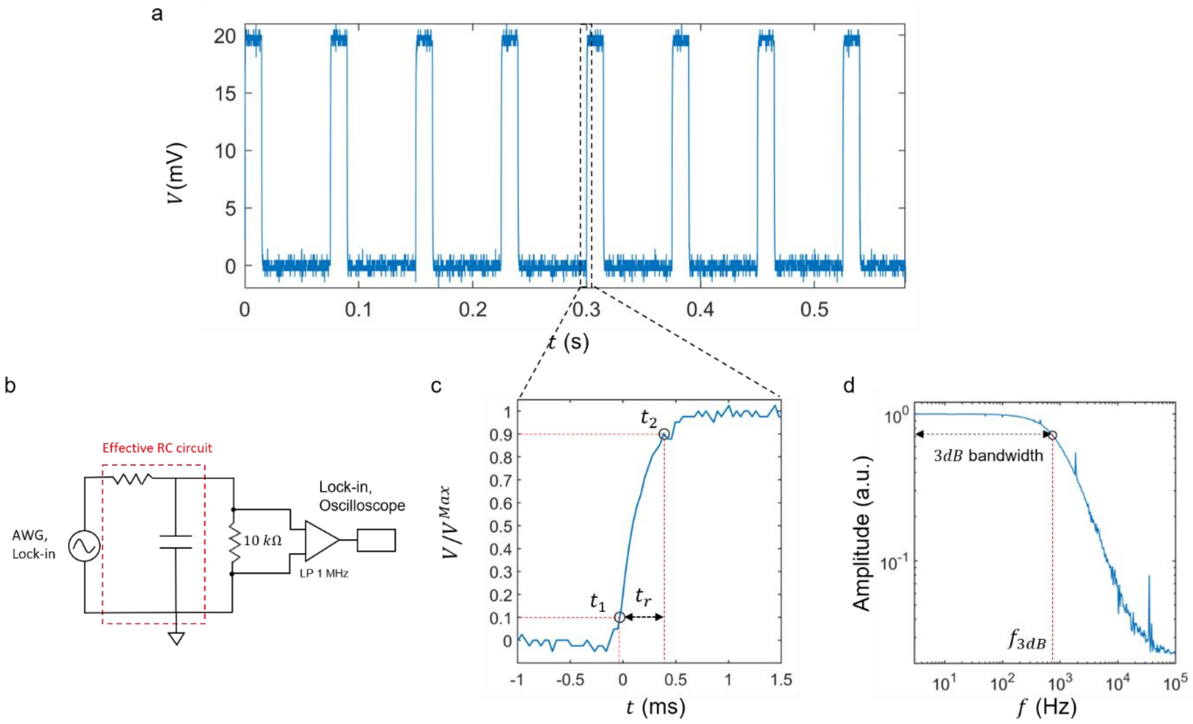

Fig. S9.| **Minimum rise time and frequency response magnitude of the electrical readout. (a)** Voltage signal measured with an oscilloscope across a 10 kilohm resistor when excited with a square wave of frequency 13.3 Hz. **(b)** Schematics of the circuit used to measure the rise time and the 3-dB bandwidth. **(c)** Zoom of the voltage traces from which we extract the rise time of the square wave pulse. **(d)** Measured frequency response magnitude of the readout.

measurement, we calculate the effective 3-dB cut-off frequency as: $f_{3dB} = 0.35/t_r$ = 830 Hz. Additionally, we directly measure the 3-dB bandwidth by applying a sinusoidal ac current to the resistor at various frequencies ranging from 3 Hz to 100 kHz using a lock-in amplifier. We measure the voltage generated across the 10 kilohm resistor with a lock-in amplifier in the same configuration (see Fig. S9d). From this measurement we can extract the 3-dB bandwidth defined as the frequency at which the initial amplitude drops by 3-dB or 0.707 of its initial value. We obtain f_{3dB} = 738 Hz. The two different measurements give compatible results.

In Fig. S10a we report an example of the photovoltage generation, V_{ph}, measured with an oscilloscope when the MATBG device is exposed to laser beam radiation of wavelength λ = 1550 nm. The measurement is performed in the configuration described in Fig. S6 using a room-temperature low-pass filter with 10 kHz cut-off. Upon photon absorption, the MATBG detector transitions to the normal state, resulting in a maximum voltage output of $V_{\mathrm{ph}}(V_{bias}) \approx V(V_{bias})$. Subsequently, the detector remains in the normal state for few ms (~ 1 ms for the trace in Fig. S10a) before the voltage bias circuit resets it to the superconducting state. It is worth noting

that our observed pulse shape differs from the typical behavior observed in conventional superconducting single-photon detectors*(3)*. In those detectors, the generated photovoltage exhibits a rapid spike followed by a slower decay with a characteristic time, τ *(5,34,51,52)*.

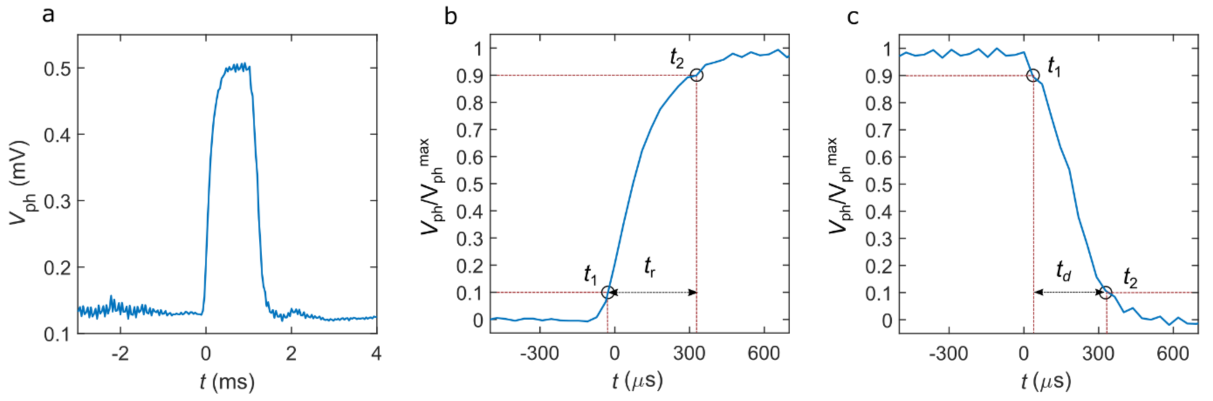

Fig. S10.| **Pulse shape, rise time and decay time for device A. (a)** Photovoltage pulse V_{ph} measured in the MATBG photodetector at V_{bias}/V_c ~ 0.989 and λ = 1550 nm with a single-shot oscilloscope. **(b)** Rise time t_r = 356 µs measured from the pulse in **(a)** which results in an overall bandwidth of the electronic readout of < 1 kHz. **(c)** The decay time t_d is similar to the rise time.

We observe that the measured pulse rise time (t_r = 356 µs) in our pulse shape (Fig. S10b) is extrinsically limited by the restricted bandwidth and compatible with the one measured with the 10 kilohm resistor. Similarly, the measured decay time is $t_d \sim t_r$. Despite the restricted bandwidth available in our experiment, we are still able to properly study the statistics of the photo-induced counts and demonstrate single-photon sensitivity by the MATBG detector.

To improve the speed of readout, a possible path forward is to design a resonator-based readout, in which the kinetic inductance is part of the resonator. When an absorbed photon generates quasiparticles inside MATBG, its kinetic inductance increases and hence suppresses the resonance frequency. This concept is similar to the kinetic inductance detector(*10*) which has proven to provide a fast readout of SPD.

Method of registering counts in the detector

In this section we show how we derive the plots shown in Fig. 3 from the raw data and detail the methods used to extract the counts of the MATBG detector. As described in the Methods section, we acquire photovoltage time traces with an analog-to-digital converter or an oscilloscope. From the raw traces (as the ones shown in Fig. 2a of the main text) we use a MATLAB script to count the number of detected events by setting a threshold (V_{ph} > 0.4 mV for device A) and a minimum distance between the clicks of 17 ms. We choose a minimum distance of 17 ms because the recovery time of the clicks varies as a function of the bias point from ~ 1 ms to ~ 17 ms. This limits the maximum measurable count rate to ~ 50 Hz. For the PCR vs P_L measurements, the minimum distance between the clicks is 13 ms.

In Fig. S11a, we present the photon count rate (PCR) as a function of V_{bias}, measured at various laser powers (from no power up to 183 $\frac{\mathrm{aW}}{\mu\mathrm{m}^2}$) as in Fig .3a of the main text. In Fig. S11 (b)-(g)

we also report the raw photovoltage time traces with and without illumination for six different bias points (vertical colored lines) from which we extracted the PCR vs. V_{bias}. In Fig. S12, we show the PCR vs. P_L for $V_{bias} = 0.995\ V_c$ as in Fig. 3b of the main text and the raw photovoltage time traces for six different laser powers.

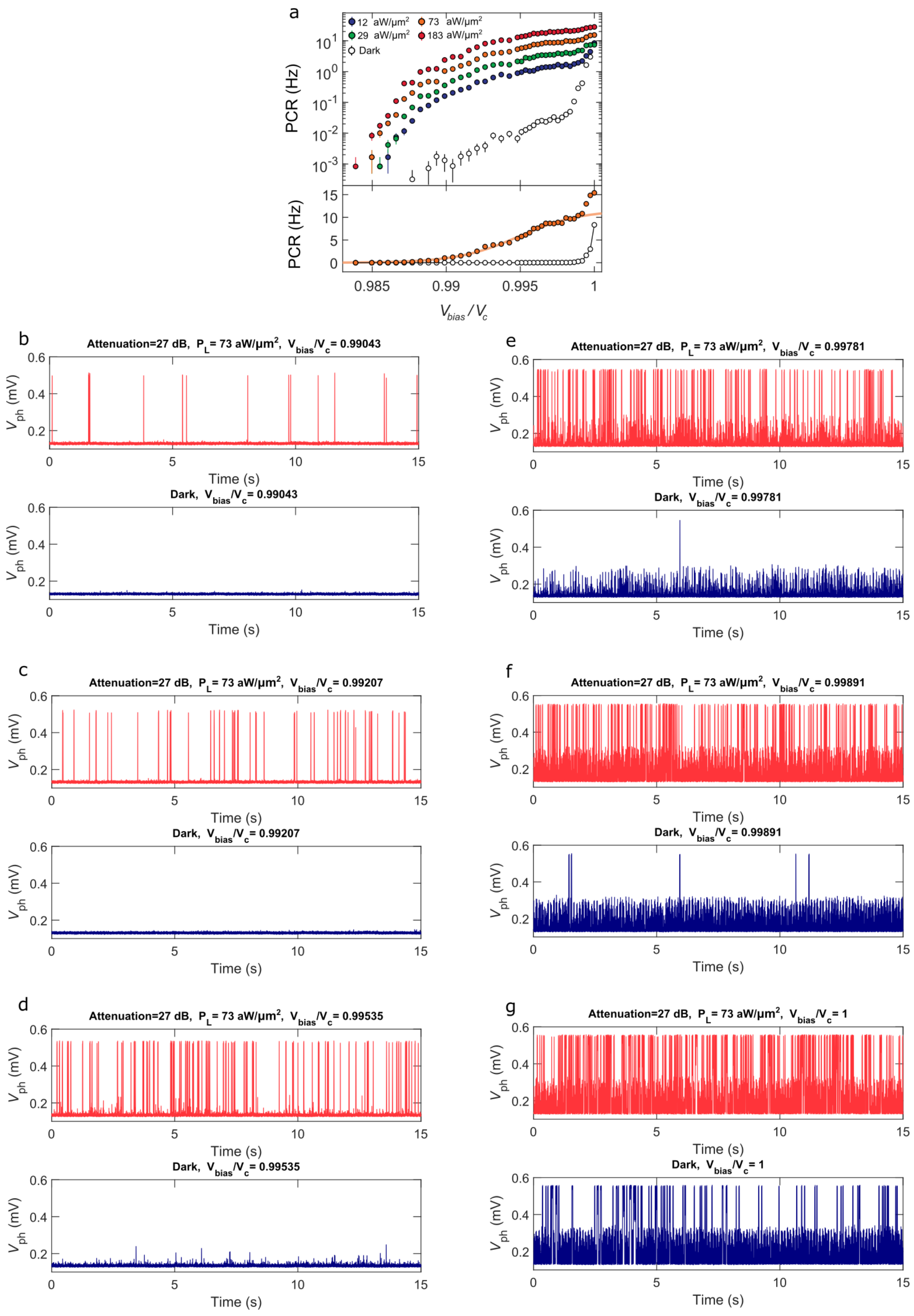

Fig. S11.| **Raw photovoltage time traces for different bias points. (a)** Photon count rate (PCR) vs. V_{bias} measured for different laser powers as in Fig. 3a. **(b)-(g)** Raw photovoltage time traces with and without 1550 nm laser-illumination for different bias points.

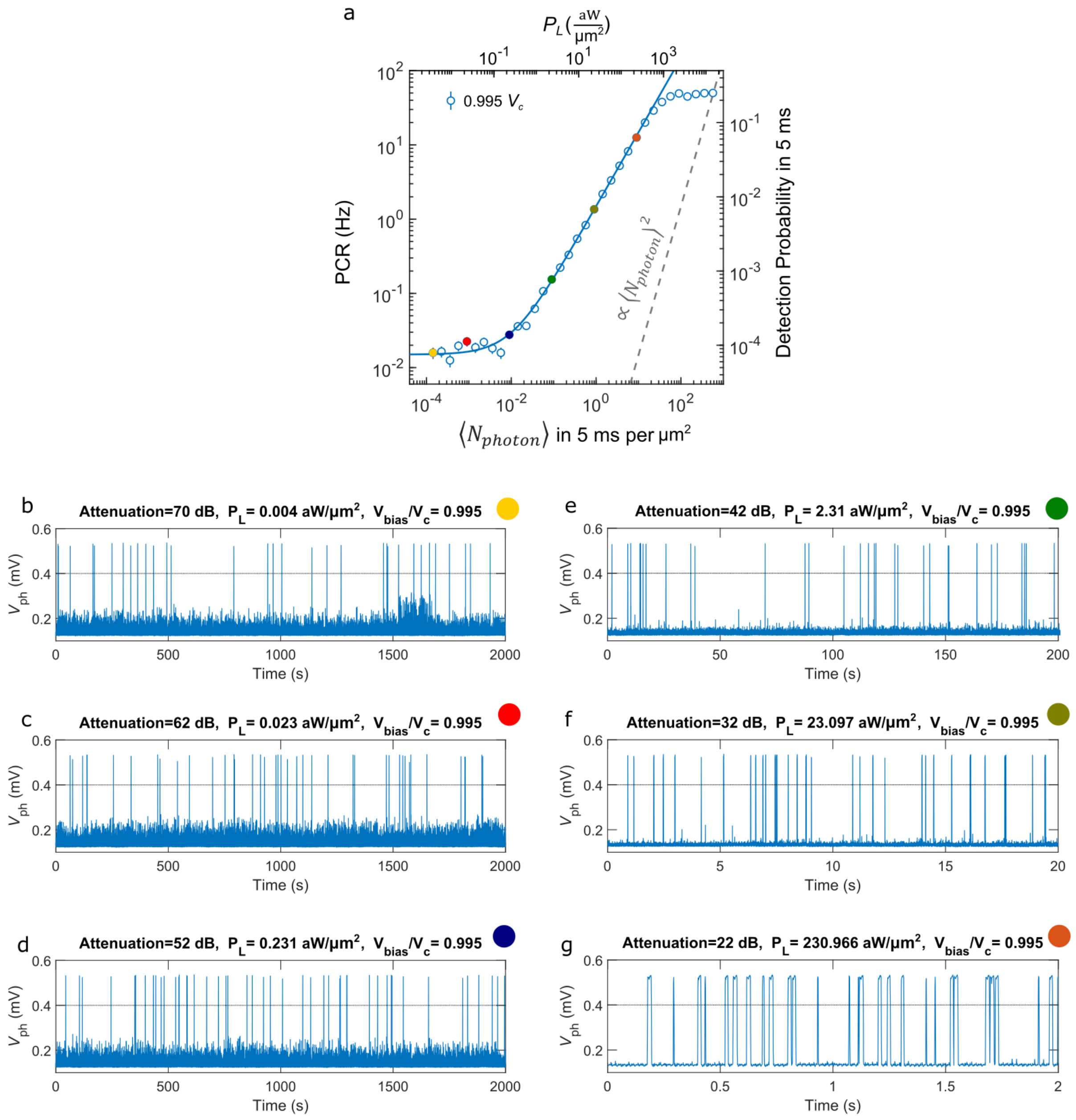

Fig. S12.| **Raw photovoltage time traces for different laser powers. (a)** Extracted photon count rate (PCR) versus laser power for V_{bias} = 0.995 V_c as shown in Fig. 3b of the main text. The colored dots are the selected laser powers for which we show the raw photovoltage time traces. **(b)-(g)** Raw photovoltage time traces measured over time for laser powers ranging over 5 orders of magnitude (laser attenuation from 70 dB to 22 dB). The black dashed line represents the threshold for counting the clicks. If the generated photovoltage is V_{ph} > 0.4 mV, we register a count in the detector. **(b)-(d)** Raw photovoltage time traces measured over 2000 second for attenuations of 70, 62 and 52 dB. In this range, the PCR (< 0.02 Hz) is not affected by the increase of laser power, meaning that the observed counts are mostly due to false positive (dark) counts. **(e)-(g)**. Raw photovoltage time traces measured over 200, 20 and 2 seconds for attenuations of 42, 32 and 22 dB respectively. In these plots we scale the time duration of the traces inversely with the laser attenuation to facilitate the counting of the 'clicks' and allow to derive the PCR by eye. In this range, we observe that the PCR scales linearly with the incident power: 42 dB (PCR ~ 0.15 Hz), 32 dB (PCR ~ 1.5 Hz) 22 dB (PCR ~ 14 Hz).

Linearity of photon counts for a highly attenuated coherent source

The light emitted by a laser source can be expressed as a coherent superposition of m-photon states, $|m\rangle$ *(13,36):*

$$|\psi\rangle = e^{-\frac{|\psi|^2}{2}} \sum_{m=0}^{\infty} \frac{\psi^m}{\sqrt{m!}} |m\rangle \qquad \text{(Eq.1)}$$

Here $|\psi^2|$ is the average photon number in the coherent state, which is directly proportional to the average photon number absorbed in the detector within a certain time duration τ, i.e. $|\psi^2|$~ $\langle N_{photon}\rangle$. The probability of detecting a m-photon state in a time window τ is then given by:

$$p(\psi|m) = e^{-|\psi|^2} \frac{(|\psi^2|)^m}{m!} \qquad \text{(Eq.2)}$$

For a highly attenuated laser source, i.e. $|\psi^2|$ << 1, the higher probability is to have either no photons or single-photons, while the probability of multi-photons is smaller: $p(\psi|m = 0) \gg p(\psi|m = 1) \gg p(\psi|m \geq 2)$. As the probability of single-photon events (m = 1) scales linearly with $|\psi^2|$, by analyzing the linear scaling of the photon count rate with $\langle N_{photon}\rangle$ in Fig. 3B, we conclude that the MATBG detector is capable of detecting single-photons.

Single-photon sensitivity with pulsed light excitation

An independent way to cross-check the single-photon sensitivity observed under CW illumination is provided by measurements with pulsed light excitation. For this purpose, we use a ~ 50 ps laser source at λ = 1550 nm with broadly tunable repetition rate, f_{RR}. The pulsed laser source allows independent control of both the number of photons carried on average by each pulse (μ) and of the frequency at which the pulses impinge on the device (inset of Fig. S13a). Having at disposal these tuning knobs, we demonstrate that the MATBG detector responds to pulses with less than one photon on average and confirm the linearity of single-photon sensitivity even under pulsed light excitation.

First, we measure the count rate at different f_{RR} spanning over several orders of magnitude from 10 Hz to 1 MHz while fixing the number of photons carried on average by each pulse, μ. Specifically, we keep $\mu = P_L A/h\nu f_{RR}$ = 0.62 < 1 fixed by tuning simultaneously f_{RR} and P_L. Here A ~ 16 µm^2 is the area between the two voltage probes. Arguably, the effective area contributing to the photoresponse is smaller than A because of the twist angle inhomogeneity and the absorption is expected to be only a few percent, implying that the μ calculated here serves as an upper limit. Having fixed μ < 1, the majority of pulses incident in the area A carry either 0 or 1 photon and the probability of a pulse carrying 2 photons is negligible.

Fig. S13a illustrates the extracted detection efficiency (defined as the ratio of counts detected per second to photons incident per second in the area A) plotted against the laser repetition rate, revealing three distinct regimes. For f_{RR} < 100 Hz the detection efficiency decreases until it reaches a plateau which persists up to f_{RR} ~ 30 kHz. After this plateau, the detection efficiency abruptly drops. In the low repetition rate regime, the count rate is dominated by the dark counts: the rate at which the pulses carrying 1 photon are absorbed is lower than the dark count rate, resulting in a detection efficiency higher than the effective one. Within the range of repetition rates where we observe a plateau, the detection efficiency remains unaffected by the

time distance between the pulses, indicating that the absorbed photon rate is smaller than the detector recovery time. This rules out steady state heating from the laser source for average powers below < 300 aW/μm^2. From the inset in Fig. S13a we observe that in this range of powers the PCR scales linearly with the average number of photons absorbed per second, confirming that the MATBG is operating as a single-photon detector. The drop of detection efficiency observed at high repetition rates is instead attributed to a saturation of the MATBG detector count rate and consistently occurs at the same average powers as in the CW experiment (> 300 aW/μm^2). Subsequently, we fix the repetition rate to 5 kHz, where the detection efficiency is independent of f_{RR}, and change the average laser power to control the number of photons carried on average by each pulse. Fig. S13b demonstrates that when the mean photon number per pulse is less than 1, the count rate evolves linearly with μ for two distinct bias voltages over several orders of magnitude. This observation further validates the single-photon sensitivity under pulsed excitation.

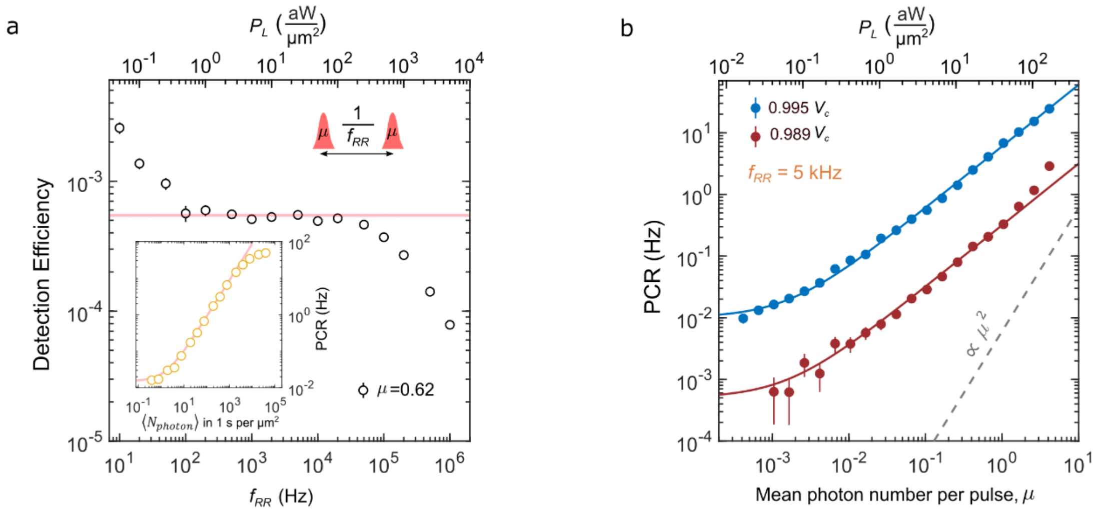

Fig. S13.| **Single-photon sensitivity with pulsed light excitation. (a)** Detection efficiency measured at fixed mean photon number per pulse μ = 0.62 and different laser repetition rates, f_{RR} for V_{bias} = 0.995 V_c. Here the detection efficiency is defined as counts detected per second over photons incident per second in the area marked by the two voltage probes (A ~ 16 μm^2). On the top x-axis the average incident power density P_L corresponding to each f_{RR}. The solid line highlights the plateau in detection efficiency observed between 100 Hz to 30 kHz. Inset: photon count rate, PCR versus the average incident photon number $\langle N_{\text{photon}} \rangle$ in 1-s time window per μm^2. The solid line is a linear fit with an offset due to dark counts. **(b)** PCR versus μ for two different bias points at a fixed f_{RR} = 5 kHz. The solid lines are linear fits (with an offset due to dark counts), showing that the PCR evolves linearly with μ.

Additional photoresponse data of device A

For completeness we report additional photoresponse data measured on device A. In Fig. S14a we plot the PCR vs. V_{bias} measured at various laser powers as in Fig. 3a of the main text but in linear scale. In the linear scale it is possible to see the sigmoidal shape with tendency to saturation. Specifically, in Fig. S14b, we plot the PCR vs. P_L in correspondence of these saturation plateaus and show that they evolve linearly with laser power ruling out an artifact form the limited bandwidth. In Fig. S14c we plot the PCR vs. P_L measured at a different bias point (V_{bias} = 0.991 V_c) than the ones reported in Fig. 3 of the main text. Even at this V_{bias} the MATBG detector shows single-photon sensitivity.

We also measure the PCR vs. P_L measured at T = 700 mK and V_{bias} = 0.996 V_c (Fig. S15) and observe a linear scaling of the PCR with P_L, demonstrating single-photon sensitivity up to this temperature.

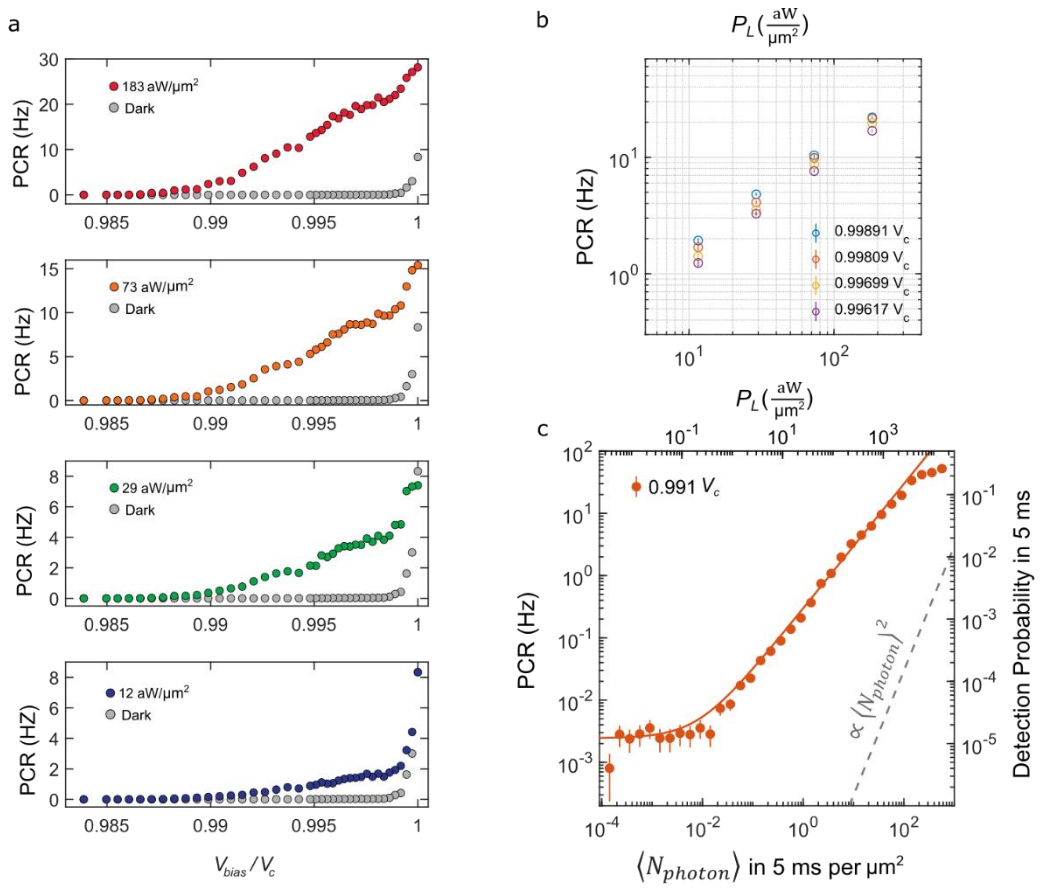

Fig. S14.| **Additional photoresponse data of device A. (a)** Extracted photon count rate (PCR) vs. V_{bias} measured at various laser powers and plotted in linear scale. **(b)** PCR vs. P_L in correspondence of the saturation plateaus ~ 0.997 V_c. **(c)** PCR vs. P_L measured at V_{bias} = 0.991 V_c. The MATBG detector shows single-photon sensitivity even at this bias point.

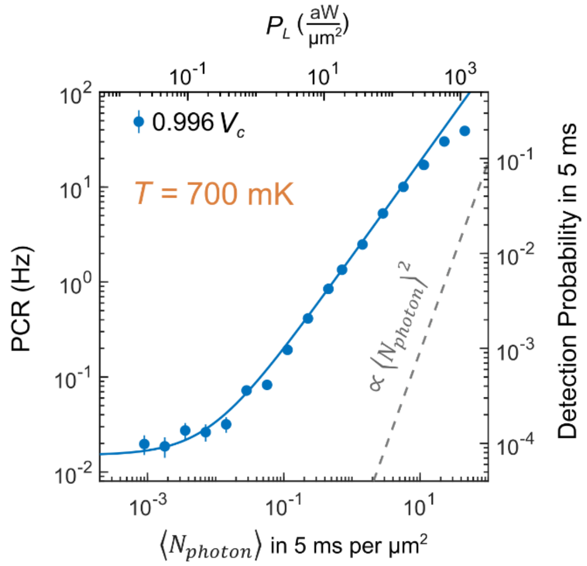

Fig. S15.| **Single-photon sensitivity at T = 700 mK.** PCR vs. average incident photon number $\langle N_{\text{photon}} \rangle$ at T = 700 mK and V_{bias} = 0.996 V_c. The solid line is a linear fit (with an offset due to dark counts) demonstrating single-photon sensitivity up to 700 mK.

Photovoltage generation and pulse shape

Here we show the pulse shapes measured for the three devices and discuss the origin of the photovoltage generated in MATBG devices. We argue that the photovoltage studied here is due to a complete breaking of the superconducting state upon absorption of a photon. In Fig. S16 we show the oscilloscope traces recorded while sweeping the bias voltage across the transition in both directions from the superconducting to normal state and vice-versa. By comparing them with the pulse shapes measured for device A, B and C (at V_{bias} = 0.989 V_c, V_{bias} = 0.991 V_c and V_{bias} = 0.9994 V_c respectively) we notice that the voltage output induced by the photons matches the voltage generated by manually sweeping the device across the transition.

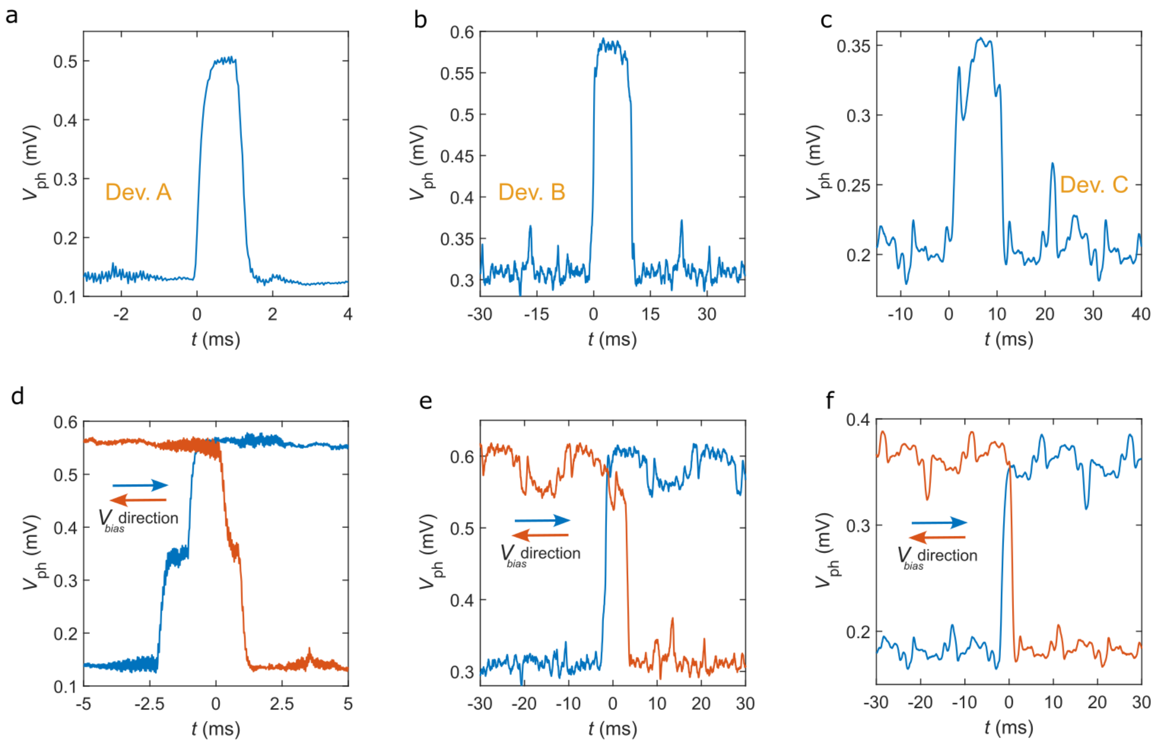

Fig. S16.| **Photovoltage generation and pulse shape for all devices. (a)-(c)** Typical pulse shape measured with a single-shot oscilloscope upon photo-absorption for device A, B and C at V_{bias} = 0.989 V_c, V_{bias} = 0.991 V_c and V_{bias} = 0.9994 V_c respectively. **(d)-(f)** Single-shot oscilloscope traces recorded while sweeping the bias voltage across the transition in both directions from the superconducting to normal state (blue) and vice-versa (red).

Photoresponse of device B

In this section we summarize the optoelectronic measurements performed on device B. As discussed in the transport characterization, device B features *I-V* curves very similar to device A but the superconducting state is not fully developed since it does not reach zero resistance (Fig. S4e). We attribute this to twist angle inhomogeneity*(44)*. In this device we measure the photovoltage time traces using the exact same setup and circuit presented above and also observe voltage spikes which increase as we increase the incident laser power. However, we observe a substantial increase of the dark count rate in device B, compared to device A. For instance, at V_{bias} ~ 0.99 V_c, device A exhibits a dark count rate of approximately 10^{-3} Hz, while device B of around 3 × 10^{-1} Hz (see Fig. S18). In SPDs, the superconducting gap typically protects the superconducting state from external excitations that turns the superconductor normal and results in dark counts. We can expect a higher dark count in device B because of the non-zero resistive state observed in the transport characterization.

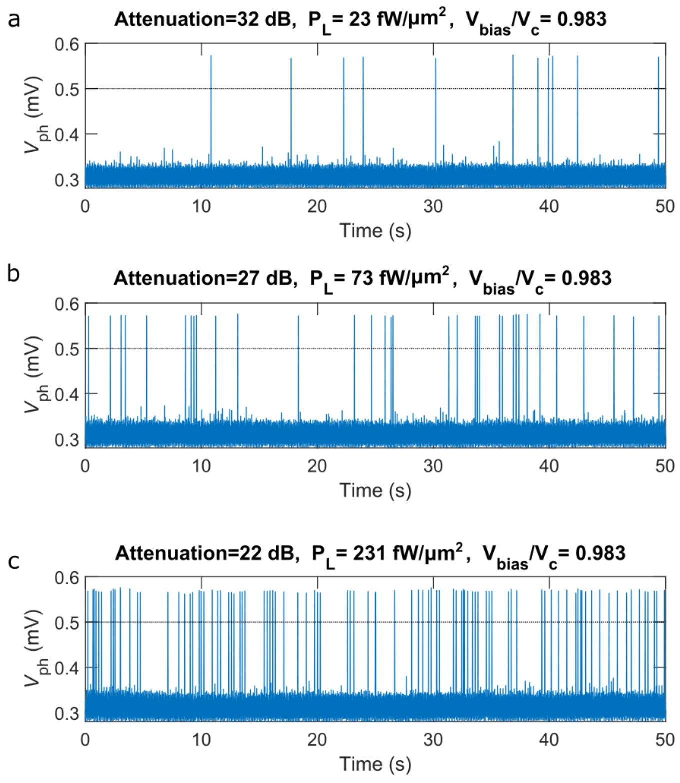

Fig. S17.| **Raw photovoltage time traces at different laser powers for device B. (a)-(c)** Raw photovoltage time traces measured over time for 3 laser powers. The black dashed line represents the threshold for counting the clicks. If the generated photovoltage is V_{ph} > 0.5 mV, we register a count in the detector.

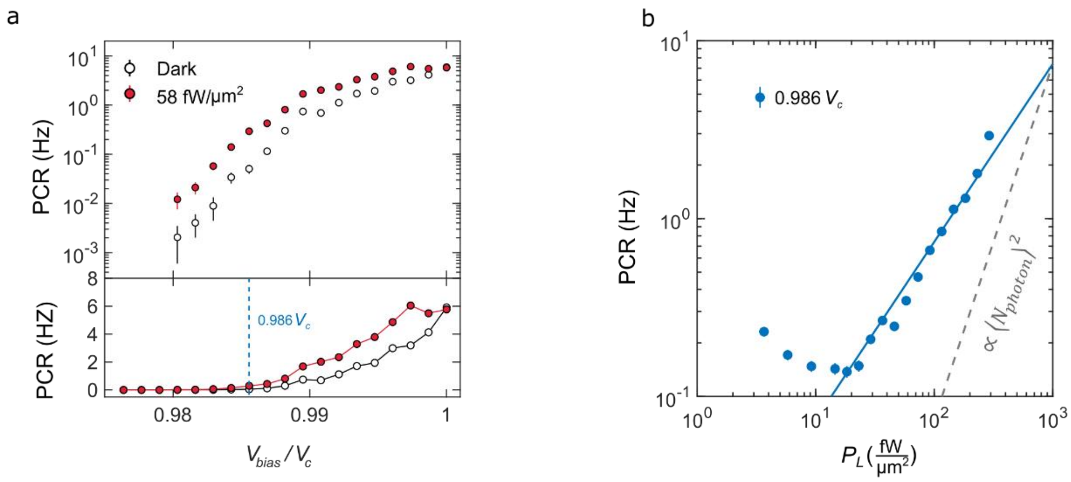

Fig. S18.| **Photoresponse of device B. (a)** Photon count rate (PCR) vs. V_{bias} measured with and without 1550 nm laser-illumination. **(b)** Extracted photon count rate PCR versus laser power for the bias points indicated by the vertical dashed line in **(b)**. The solid line is a linear fit.

Photoresponse of device C

In this section we summarize the optoelectronic measurements performed on device C. Device C features different *I-V* characteristics than device A and B because it has a smaller hysteresis loop (~ 2-3 nA). In addition, while device A and B feature a sharp transition from superconducting to normal state, device C shows a smooth transition. In Fig. S19 we show the light-induced switching events recorded in device C and in Fig. S19 we summarize the analysis performed on these photovoltage time traces.

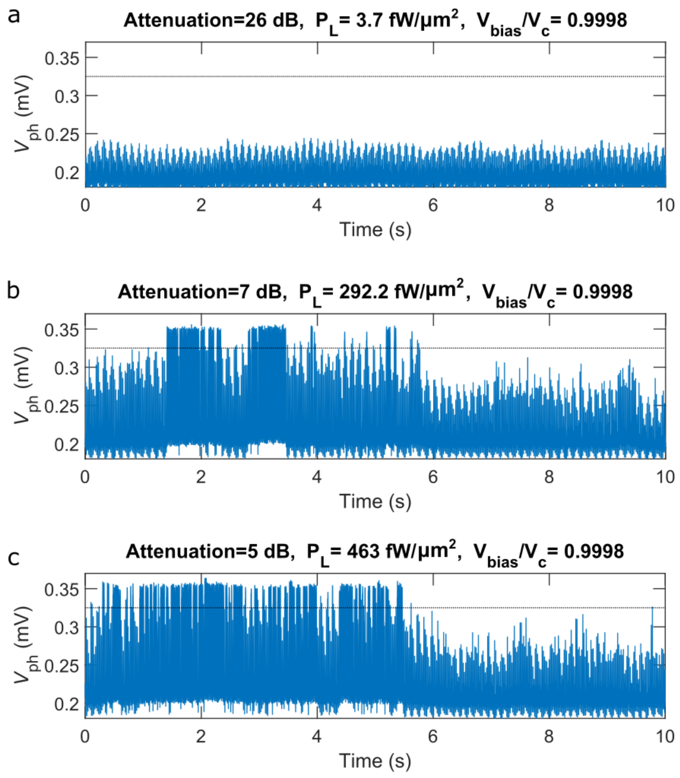

Fig. S19.| **Raw photovoltage time traces at different laser powers for device C. (a-c)** Raw photovoltage time traces measured over time for 3 laser powers. The black dashed line represents the threshold for counting the clicks. If the generated photovoltage is $V_{ph} > 0.325$ mV, we register a count in the detector.

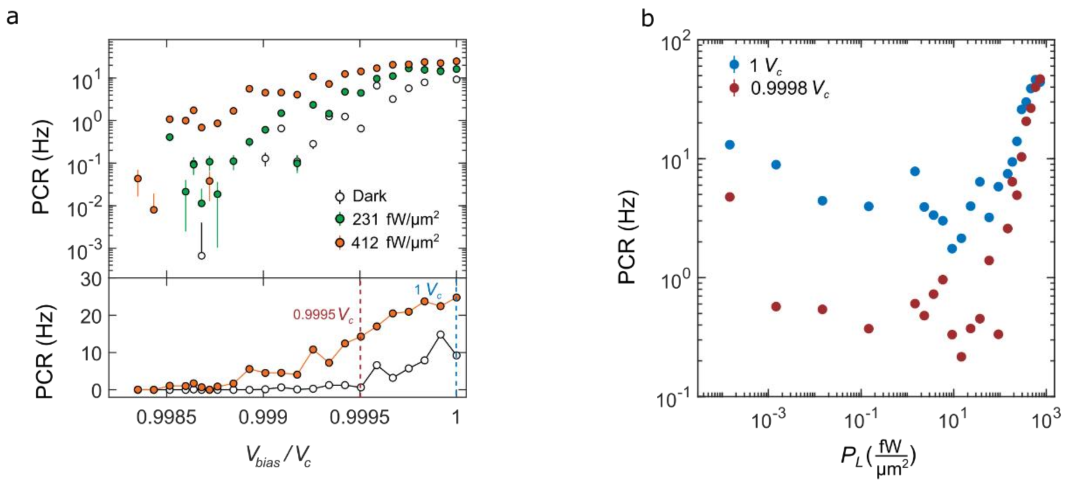

Fig. S20.| **Photoresponse of device C. (a)** Photon count rate (PCR) vs. V_{bias} measured with and without 1550 nm laser-illumination. **(b)** Extracted photon count rate PCR versus laser power for the bias points indicated by the vertical dashed lines in **(b)**.

REFERENCES AND NOTES

1. G. N. Gol'tsman, O. Okunev, G. Chulkova, A. Lipatov, A. Semenov, K. Smirnov, B. Voronov, A. Dzardanov, C. Williams, R. Sobolewski, Picosecond superconducting single-photon optical detector. *Appl. Phys. Lett.* **79**, 705–707 (2001).

2. A. D. Semenov, G. N. Gol'tsman, A. A. Korneev, Quantum detection by current carrying superconducting film. *Physica C* **351**, 349–356 (2001).

3. D. V. Morozov, A. Casaburi, R. H. Hadfield, Superconducting photon detectors. *Contemp. Phys.* **62**, 69–91 (2021).

4. B. Cabrera, R. M. Clarke, P. Colling, A. J. Miller, S. Nam, R. W. Romani, Detection of single infrared, optical, and ultraviolet photons using superconducting transition edge sensors. *Appl. Phys. Lett.* **73**, 735–737 (1998).

5. K. D. Irwin, G. C. Hilton, Transition-edge sensors. *Topics Appl. Phys.* **99**, 63–149 (2005).

6. I. Holzman, Y. Ivry, Superconducting nanowires for single-photon detection: Progress, challenges, and opportunities. *Adv. Quant. Technol.* **2**, 1800058 (2019).

7. F. Marsili, V. B. Verma, J. A. Stern, S. Harrington, A. E. Lita, T. Gerrits, I. Vayshenker, B. Baek, M. D. Shaw, R. P. Mirin, S. W. Nam, Detecting single infrared photons with 93% system efficiency. *Nat. Photonics* **7**, 210–214 (2013).

8. V. B. Verma, B. Korzh, A. B. Walter, A. E. Lita, R. M. Briggs, M. Colangelo, Y. Zhai, E. E. Wollman, A. D. Beyer, J. P. Allmaras, H. Vora, D. Zhu, E. Schmidt, A. G. Kozorezov, K. K. Berggren, R. P. Mirin, S. W. Nam, M. D. Shaw, Single-photon detection in the mid-infrared up to 10 μm wavelength using tungsten silicide superconducting nanowire detectors. *APL Photonics* **6**, 056101 (2021).

9. B. S. Karasik, S. V. Pereverzev, A. Soibel, D. F. Santavicca, D. E. Prober, D. Olaya, M. E. Gershenson, Energy-resolved detection of single infrared photons with $\lambda = 8$ μm using a superconducting microbolometer. *Appl. Phys. Lett.* **101**, 052601 (2012).

10. P. K. Day, H. G. LeDuc, B. A. Mazin, A. Vayonakis, J. Zmuidzinas, A broadband superconducting detector suitable for use in large arrays. *Nature* **425**, 817–821 (2003).

11. E. D. Walsh, D. K. Efetov, G.-H. Lee, M. Heuck, J. Crossno, T. A. Ohki, P. Kim, D. Englund, K. C. Fong, Graphene-based Josephson-junction single-photon detector. *Phys. Rev. Appl.* **8**, 024022 (2017).

12. G. H. Lee, D. K. Efetov, W. Jung, L. Ranzani, E. D. Walsh, T. A. Ohki, T. Taniguchi, K. Watanabe, P. Kim, D. Englund, K. C. Fong, Graphene-based Josephson junction microwave bolometer. *Nature* **586**, 42–46 (2020).

13. E. D. Walsh, W. Jung, G. H. Lee, D. K. Efetov, B.-I. Wu, K.-F. Huang, T. A. Ohki, T. Taniguchi, K. Watanabe, P. Kim, D. Englund, K. C. Fong, Josephson junction infrared single-photon detector. *Science* **372**, 409–412 (2021).

14. D. J. Benford, S. H. Mosley, Cryogenic detectors for infrared astronomy: The single aperture far-infrared (SAFIR) observatory. *Nucl. Instrum. Methods Phys. Res. Sect. A* **520**, 379–383 (2004).

15. E. Knill, R. Laflamme, G. J. Milburn, A scheme for efficient quantum computation with linear optics. *Nature* **409**, 46–52 (2001).

16. J. A. Lau, V. B. Verma, D. Schwarzer, A. M. Wodtke, Superconducting single-photon detectors in the mid-infrared for physical chemistry and spectroscopy. *Chem. Soc. Rev.* 921–941 (2023).

17. P. Seifert, J. R. D. Retamal, R. L. Merino, H. H. Sheinfux, J. N. Moore, M. A. Aamir, T. Taniguchi, K. Watanabe, K. Kadowaki, M. Artiglia, M. Romagnoli, D. K. Efetov, A high-T_c van der Waals superconductor based photodetector with ultra-high responsivity and nanosecond relaxation time. *2D Mater.* **8**, 035053 (2021).

18. R. L. Merino, P. Seifert, J. Duran Retamal, R. Mech, T. Taniguchi, K. Watanabe, K. Kadowaki, R. H. Hadfield, D. K. Efetov, Two-dimensional cuprate nanodetector with single telecom photon sensitivity at T = 20 K. *2D Mater.* **10**, 021001 (2023).

19. I. Charaev, D. A. Bandurin, A. T. Bollinger, I. Y. Phinney, I. Drozdov, M. Colangelo, B. A. Butters, T. Taniguchi, K. Watanabe, X. He, O. Medeiros, I. Božović, P. Jarillo-Herrero, K. K. Berggren, Single-photon detection using high-temperature superconductors. *Nat. Nanotechnol.* **18**, 343–349 (2023).

20. G. J. Orchin, D. De Fazio, A. Di Bernardo, M. Hamer, D. Yoon, A. R. Cadore, I. Goykhman, K. Watanabe, T. Taniguchi, J. W. A. Robinson, R. V. Gorbachev, A. C. Ferrari, R. H. Hadfield, Niobium diselenide superconducting photodetectors. *Appl. Phys. Lett.* **114**, 251103 (2019).

21. R. Kokkoniemi, J.-P. Girard, D. Hazra, A. Laitinen, J. Govenius, R. E. Lake, I. Sallinen, V. Vesterinen, M. Partanen, K. Y. Tan, P. Hakonen, M. Möttönen, Bolometer operating at the threshold for circuit quantum electrodynamics. *Nature* **586**, 47–51 (2020).

22. Y. Cao, V. Fatemi, S. Fang, K. Watanabe, T. Taniguchi, E. Kaxiras, P. Jarillo-Herrero, Unconventional superconductivity in magic-angle graphene superlattices. *Nature* **556**, 43–50 (2018).

23. R. Bistritzer, A. H. MacDonald, Moiré bands in twisted double-layer graphene. *Proc. Natl. Acad. Sci. U.S.A.* **108**, 12233–12237 (2011).

24. J. M. Park, Y. Cao, K. Watanabe, T. Taniguchi, P. Jarillo-Herrero, Tunable strongly coupled superconductivity in magic-angle twisted trilayer graphene. *Nature* **590**, 249–255 (2021).

25. J. M. Park, Y. Cao, L.-Q. Xia, S. Sun, K. Watanabe, T. Taniguchi, P. Jarillo-Herrero, Robust superconductivity in magic-angle multilayer graphene family. *Nat. Mater.* **21**, 877–883 (2022).

26. H. Zhou, T. Xie, T. Taniguchi, K. Watanabe, A. F. Young, Superconductivity in rhombohedral trilayer graphene. *Nature* **598**, 434–438 (2021).

27. H. Zhou, L. Holleis, Y. Saito, L. Cohen, W. Huynh, C. L. Patterson, F. Yang, T. Taniguchi, K. Watanabe, A. F. Young, Isospin magnetism and spin-polarized superconductivity in Bernal bilayer graphene. *Science* **375**, 774–778 (2022).

28. P. Seifert, X. Lu, P. Stepanov, J. R. D. Retamal, J. N. Moore, K. C. Fong, A. Principi, D. K. Efetov, Magic-angle bilayer graphene nano-calorimeters: Towards broadband, energy-resolving single photon detection. *Nano Lett.* **20**, 3459–3464 (2020).

29. F. J. Gonzalez, M. S. Lodge, M. Ishigami, R. A. Klemm, A. Rathod, K. L. Lina, A. C. Bowman, F. Hernandez, C. J. Fredricksen, C. Cariker, R. E. Peale, Antenna-coupled graphene Josephson-junction terahertz detector. *MRS Adv.* **8**, 148–151 (2023).

30. G. Di Battista, P. Seifert, K. Watanabe, T. Taniguchi, K. C. Fong, A. Principi, D. K. Efetov, Revealing the thermal properties of superconducting magic-angle twisted bilayer graphene. *Nano Lett.* **22**, 6465–6470 (2022).

31. Y. Cao, D. Rodan-Legrain, J. M. Park, N. F. Q. Yuan, K. Watanabe, T. Taniguchi, R. M. Fernandes, L. Fu, P. Jarillo-Herrero, Nematicity and competing orders in superconducting magic-angle graphene. *Science* **372**, 264–271 (2021).

32. W. J. Skocpol, M. R. Beasley, M. Tinkham, Self-heating hotspots in superconducting thin-film microbridges. *J. Appl. Phys.* **45**, 4054–4066 (1974).

33. M. Tinkham, J. U. Free, C. N. Lau, N. Markovic, Hysteretic $I - V$ curves of superconducting nanowires. *Phys. Rev. B* **68**, 134515 (2003).

34. A. J. Kerman, J. K. W. Yang, R. J. Molnar, E. A. Dauler, K. K. Berggren, Electrothermal feedback in superconducting nanowire single-photon detectors. *Phys. Rev. B* **79**, 100509 (2009).

35. K. D. Irwin, An application of electrothermal feedback for high resolution cryogenic particle detection. *Appl. Phys. Lett.* **66**, 1998 (1995).

36. R. Loudon, *Quantum Theory of Light* (Oxford University Press, ed. 3, 2000).

37. B. Baek, A. E. Lita, V. B. Verma, S. W. Nam, Superconducting a-W_xSi_{1-x} nanowire single-photon detector with saturated internal quantum efficiency from visible to 1850 nm. *Appl. Phys. Lett.* **98**, 251105 (2011).

38. O. Svelto, *Principles of Lasers* (Springer, ed. 5, 2012).

39. R. H. Hadfield, Single-photon detectors for optical quantum information applications. *Nat. Photonics* **3**, 696–705 (2009).

40. T. Yamashita, S. Miki, W. Qiu, M. Fujiwara, M. Sasaki, Z. Wang, Temperature dependent performances of superconducting nanowire single-photon detectors in an ultralow-temperature region. *Appl. Phys. Express* **3**, 102502 (2010).

41. F. C. Wellstood, C. Urbina, J. Clarke, Hot-electron effects in metals. *Phys. Rev. B* **49**, 5942–5955 (1994).

42. Y. Cao, V. Fatemi, A. Demir, S. Fang, S. L. Tomarken, J. Y. Luo, J. D. Sanchez-Yamagishi, K. Watanabe, T. Taniguchi, E. Kaxiras, R. C. Ashoori, P. Jarillo-Herrero, Correlated insulator behaviour at half-filling in magic-angle graphene superlattices. *Nature* **556**, 80–84 (2018).

43. M. Oh, K. P. Nuckolls, D. Wong, R. L. Lee, X. Liu, K. Watanabe, T. Taniguchi, A. Yazdani, Evidence for unconventional superconductivity in twisted bilayer graphene. *Nature* **600**, 240–245 (2021).

44. A. Uri, S. Grover, Y. Cao, J. A. Crosse, K. Bagani, D. Rodan-Legrain, Y. Myasoedov, K. Watanabe, T. Taniguchi, P. Moon, M. Koshino, P. Jarillo-Herrero, E. Zeldov, Mapping the twist-angle disorder and Landau levels in magic-angle graphene. *Nature* **581**, 47–52 (2020).

45. T. Benschop, T. A. de Jong, P. Stepanov, X. Lu, V. Stalman, S. J. van der Molen, D. K. Efetov, M. P. Allan, Measuring local moiré lattice heterogeneity of twisted bilayer graphene. *Phys. Rev. Res.* **3**, 013153 (2021).

46. W. N. Kang, C. U. Jung, K. H. P. Kim, M.-S. Park, S. Y. Lee, H.-J. Kim, E.-M. Choi, K. H. Kim, M.-S. Kim, S.-I. Lee, Hole carrier in MgB_2 characterized by Hall measurements. *Appl. Phys. Lett.* **79**, 982–984 (2001).

47. A. Gozar, G. Logvenov, L. F. Kourkoutis, A. T. Bollinger, L. A. Giannuzzi, D. A. Muller, I. Bozovic, High-temperature interface superconductivity between metallic and insulating copper oxides. *Nature* **455**, 782–785 (2008).

48. N. A. Saveskul, N. A. Titova, E. M. Baeva, A. V. Semenov, A. V. Lubenchenko, S. Saha, H. Reddy, S. I. Bogdanov, E. E. Marinero, V. M. Shalaev, A. Boltasseva, V. S. Khrapai, A. I. Kardakova, G. N. Goltsman, Superconductivity behavior in epitaxial TiN films points to surface magnetic disorder. *Phys. Rev. Appl.* **12**, 054001 (2019).

49. A. Stangl, A. Palau, G. Deutscher, X. Obradors, T. Puig, Ultra-high critical current densities of superconducting $YBa_2Cu_3O_{7-\delta}$ thin films in the overdoped state. *Sci. Rep.* **11**, 8176 (2021).

50. H. Ge, Y. R. Jin, X.-H. Song, High quality NbTiN films fabrication and rapid thermal annealing investigation. *Chinese Phys. B* **28**, 077402 (2019).

51. A. J. Annunziata, O. Quaranta, D. F. Santavicca, A. Casaburi, L. Frunzio, M. Ejrnaes, M. J. Rooks, R. Cristiano, S. Pagano, A. Frydman, D. E. Prober, Reset dynamics and latching in niobium superconducting nanowire single-photon detectors. *J. Appl. Phys.* **108**, 084507 (2010).

52. X. Du, D. E. Prober, H. Vora, C. B. McKitterick, Graphene-based bolometers. *Graphene 2D Mater.* **1**, 1–22 (2014).

53. G. F. Burkhard, E. T. Hoke, M. D. McGehee, Accounting for interference, scattering, and electrode absorption to make accurate internal quantum efficiency measurements in organic and other thin solar cells. *Adv. Mater.* **22**, 3293–3297 (2010).

54. M. Furchi, A. Urich, A. Pospischil, G. Lilley, K. Unterrainer, H. Detz, P. Klang, A. M. Andrews, W. Schrenk, G. Strasser, T. Mueller, Microcavity-integrated graphene photo-detector. *Nano Lett.* **12**, 2773–2777 (2012).

55. D. K. Efetov, R.-J. Shiue, Y. Gao, B. Skinner, E. D. Walsh, H. Choi, J. Zheng, C. Tan, G. Grosso, C. Peng, J. Hone, K. C. Fong, D. Englund, Fast thermal relaxation in cavity-coupled graphene bolometers with a Johnson noise read-out. *Nat. Nanotechnol.* **13**, 797–801 (2018).
